# Adverse risk factor trends limit gains in coronary heart disease mortality in Barbados: 1990-2012

**DOI:** 10.1371/journal.pone.0215392

**Published:** 2019-04-17

**Authors:** N. P. Sobers, N. Unwin, T. A. Samuels, S. Capewell, M. O’Flaherty, J. A. Critchley

**Affiliations:** 1 Faculty of Medical Sciences, The University of the West Indies, Bridgetown, Barbados; 2 UKCRC Centre for Diet and Activity Research (CEDAR), MRC Epidemiology Unit, University of Cambridge School of Clinical Medicine, Cambridge, United Kingdom; 3 George Alleyne Chronic Disease Research Centre, Caribbean Institute for Health Research, The University of the West Indies, Bridgetown, Barbados; 4 Department of Public Health and Policy, University of Liverpool, Liverpool, United Kingdom; 5 Population Health Research Institute, St. George’s, University of London, London, United Kingdom; University of Mississippi Medical Center, UNITED STATES

## Abstract

**Background:**

Although most countries face increasing population levels of obesity and diabetes their effect on coronary heart disease (CHD) mortality has not been often studied in small island developing states (SIDs) where obesity rates are among the highest in the world. We estimated the relative contributions of treatments and cardiovascular risk factors to the decline in CHD mortality from 1990 to 2012 in the Caribbean island, Barbados.

**Methods:**

We used the IMPACT CHD mortality model to estimate the effect of increased coverage of effective medical/surgical treatments and changes in major CHD risk factors on mortality trends in 2012 compared with 1990. We calculated deaths prevented or postponed (DPPs) for each model risk factor and treatment group. We obtained data from WHO Mortality database, population denominators from the Barbados Statistical Service stratified by 10-year age group (ages 25–34 up to 85 plus), population-based risk factor surveys, Global Burden of Disease and Barbados’ national myocardial infarction registry. Monte Carlo probabilistic sensitivity analysis was performed.

**Results:**

In 1990 the age-standardized CHD mortality rate was 109.5 per 100,000 falling to 55.3 in 2012. Implementation of effective treatment accounted for 56% DPPs (95% (Uncertainty Interval (UI) 46%, 68%), mostly due to the introduction of treatments immediately after acute myocardial infarction (AMI) (14%) and unstable angina (14%). Overall, risk factors contributed 19% DPPs (95% UI 6% to 34%) mostly attributed to decline in cholesterol (18% DPPs, 95% UI 12%, 26%). Adverse trends in diabetes: 14% additional deaths(ADs) 95% UI 8% to 21% ADs) and BMI (2% ADs 95%UI 0 to 5% ADs) limited potential for risk factor gains.

**Conclusions:**

Given the significant negative impact of obesity/diabetes on mortality in this analysis, research that explores factors affecting implementation of evidenced-based preventive strategies is needed. The fact that most of the decline in CHD mortality in Barbados was due to treatment provides an example for SIDs about the advantages of universal access to care and treatment.

## Introduction

Coronary heart disease (CHD) is the number one cause of death in the sub-region of the Americas known as the Caribbean[1]. The Caribbean consists of 32 territories and small island developing states (SIDS), approximately half of which are classified as lower and/or middle income. Although most countries globally face increasing population levels of obesity, diabetes and non-communicable diseases (NCDs), rates in SIDs are among the highest in the world[2–4]. Many developed countries have been able to achieve decreases in CHD mortality over the past three to four decades[5]. However, the picture is mixed among the nations of the Caribbean; countries such as St. Lucia, St. Vincent and Grenadines and Guyana have all recorded increases in age adjusted CHD mortality, while Barbados and Trinidad and Tobago have reported decreases over the period 1995 to 2008[6].

The factors underlying the differential rates and direction of change in CHD mortality among countries in the Caribbean are not known. Broadly there are two categories of explanation: differences in trends in the coverage of effective medical and surgical treatments and differences in trends in risk factors. In the United States, for example, previous work has suggested that around 47% of the decline in CHD mortality is attributable to improvements in treatment and 44% to trends in risk factors[7].

The cardiovascular policy model, IMPACT, has been used in England, Finland, United States and more recently in Syria and the West Bank to determine the proportion of change in CHD mortality that is due to changes in risk factor and improvements in specific treatment[8–10]. In this paper we seek to explain the trends in CHD mortality using the IMPACT model for one Caribbean island (Barbados) for the period 1990–2012. Barbados, in addition to being the location of several population-based surveys over the past decades, has the only active Non-Communicable Disease (NCD) surveillance registry in the English-speaking Caribbean and a recent population-based risk factor survey. The island was therefore chosen because of ease of access to the data to populate the model, but with the intention that the process could be used as an exemplar for other countries in the region. This is the first use of the model in a predominantly African origin population.

## Methods

### Description of IMPACT

To explain the changes in cardiovascular mortality in Barbados between 1990 and 2012 we used the IMPACT coronary heart disease (CHD) mortality model which has been applied previously applied in over 20 countries and validated against historical data in the United Kingdom. [8, 11–13]. The IMPACT model is used to estimate the number of CHD deaths prevented or postponed by each specific cardiac intervention, or risk factor decline. It incorporates all major cardiovascular risk factors, including systolic blood pressure, mean cholesterol concentration, diabetes, smoking, physical inactivity, overweight, and obesity, plus all standard treatments for CHD and heart failure (S1 Table)[14]. The details of the methods of this model have been explained previously[14]. Data in S1 and S2 Tables provide details of the coefficients and relative risks used in the model. Here we present details relevant to the Barbados study.

### Setting

Barbados is an island of 166 square miles and a population of 277 821 (according to 2010 census data), comprising 92.4%, 2.7%, 3.1% and 1.8% who self-identify as black, white, mixed race and other respectively(9). Barbados’ health care system is financed through general taxation funds provided through government budgetary allocations. Health care services can be obtained free at the point of delivery through a system of twelve outpatient primary care centres (polyclinics) and one 600 bed tertiary care facility. Citizens also have the option of paying out-of-pocket or using private health insurance at various private primary care clinics.

Barbados has had a series of well-designed population-based studies providing sound data for review and analysis. Studies such as the Barbados Eye Study (1989–1991)[15, 16], Study of Health and Wellbeing of the Elderly (1999–2000)[17], the population-based Risk factor STEPS Survey(2007) and Health of the Nation studies (2011–2013) [18] have been conducted. Barbados is home to an active multi-disease register [19].

### Population demographic changes and mortality

Information on population demographic changes, including numbers by 10-year age and sex groups, was obtained from the Barbados Statistical Service (S3 Table). Data on deaths from CHD were obtained from the mortality database of the World Health Organization (WHO) which contained figures submitted by the local ministry through the Pan American Health Organization (PAHO). For this model, we used data for persons aged 25 years and above. Given the small population size in Barbados (277,668 in 2012 and 260,491 in 1990) and small number (and thus inherent instability) of deaths per year from CHD, the number of deaths entered the model was calculated as a 3-year average from the years 1989, 1990 and 1991 (for the base year) and 2010, 2011 and 2012 (the final year). We calculated the number of deaths from CHD that would have been expected in 2012 if the mortality rates in 1990 had remained unchanged by multiplying the age-specific mortality rates for 1990 by the population for each 10-year age stratum in the year 2012. There were 10-year age and sex strata, from 25 to 84 years, plus two strata (one for men, one for women) above the age of 85. Population ageing between 1990 and 2012 was taken into account by applying the age and sex specific rates from 1990 to the 2012 population by these strata. Subtracting the number of deaths observed in 2012 from the number expected based on the 1990 population then yielded the drop in the number of deaths (prevented or postponed) in 2012 for the model to explain.

### Sources of data for cardiovascular risk factors

In 1990, there was no single nationally representative survey which provided the range of data needed for the model, thus data from the Global Burden of Disease (GBD) study were used. The GBD estimates for 1990 were based on several national and community-based surveys conducted in Barbados [20–22]. Risk factor data for 2012 were taken from a population based, nationally representative survey conducted in 2012 and known as the Health of the Nation (HotN)(18). Data on fruit and vegetable consumption and physical inactivity in 1990 were unavailable from GBD. For these two items, we assumed that the 1990 values were the same as those from the 2007 Barbados STEPS survey[23]. The limitations of this assumption are acknowledged and addressed in sensitivity analyses.

### CHD patients and uptake of cardiovascular-related treatments

From the Barbados National Registry for Chronic Non-communicable Disease (the BNR)[19], we obtained acute myocardial infarction (AMI) incidence as well as MI hospital admissions and case fatality rates for 2012 (S4 Table). Numbers of patients with unstable angina and heart failure admitted to the island’s lone tertiary care public hospital (Queen Elizabeth Hospital (QEH)) were obtained from the Ministry of Health.

We used data from the BNR for the years 2011–2014 to calculate the medication uptakes within the first 24 hours as well as on discharge. Medications recorded were Aspirin, Angiotensin-Converting Enzyme (ACE) Inhibitors/Angiotensin Receptor Blockers (ARBs), Beta-blockers, Thrombolytics and In-hospital Cardiopulmonary Resuscitation (S4 Table). Since there was no published or raw data available to inform the treatment of heart failure and angina in the hospital and the community, a retrospective chart review (n = 386) of patients being treated during the years 2010 to 2014 was conducted at QEH and six of the twelve government-owned primary health care centres. The chart review provided data on the proportions of angina and heart failure patients receiving medical and surgical treatments and estimates of patients receiving Coronary Artery-Bypass Grafting (CABG) and Percutaneous Coronary Intervention (PCI).

### Qualitative methods used to inform the model

Documentary analysis was conducted by reviewing all Chief Medical Officer (CMO) Reports and Barbados National Drug Formularies (BNDF) printed during the period 1988 to 2012. The BNDF provided information on the availability of relevant drug categories such as beta-blockers, aspirin, statins and ACE inhibitors in the public sector. The CMO reports provided information on policy changes that may have affected availability of drugs and services such as the policy of the Barbados Drug Service to make specific classes of drugs free at the point of delivery to all residents of the country.

To obtain further information on the availability of major treatment interventions, such as CABG, PCI, cardio-pulmonary resuscitation (CPR) and cardiac rehabilitation, semi-structured interviews were conducted. A purposive sample of eight health care professionals (cardiologists, emergency physicians, family practitioners) operating in the island during the late 1980s and early 1990s was chosen. The life grid approach which has been used in epidemiological studies involving dating and recall of temporally distant exposures[24–26], required that participants remember what was occurring in their personal and professional lives in the year 1990. The interview guide can be found in S1 Appendix. Data from the interviews were analysed and in cases where there was significant disagreement amongst participants about a particular question, interviewees were asked to review their initial answer in the light of what has been proposed by others. This attempt to reach consensus amongst experts is characteristic of the Delphi process. A full explanation of the documentary analysis and semi-structured interview methodology is available in S2 Appendix.

### Estimating deaths prevented or postponed (DPPs) attributed to risk factors

Using the IMPACT model we estimated the number of DPPs related to changes in the prevalence of cardiovascular risk factors in the population. The risk factors considered were cigarette smoking, total cholesterol, systolic blood pressure, body mass index, diabetes, physical inactivity and mean fruit and vegetable consumption per day. The number of DPPs from changes in these risk factors was estimated using two approaches. For those risk factors measured as continuous variables (blood pressure, total cholesterol and BMI), the regression β coefficients approach was used to quantify the population mortality impact. These β coefficients were obtained from international pooling studies (S2 Table). The DPPs as a result of the change in the prevalence of or mean value for each of these risk factors was estimated as the product of three variables: the number of deaths from CHD in 1990 (the initial year), the subsequent reduction in that risk factor, and the regression coefficient (adjusted for confounders) quantifying the change in mortality from CHD per unit of absolute change in the risk factor. This calculation was repeated for each 10-year age-sex group.

The second approach, population-attributable risk fraction, was employed for categorical variables—diabetes, physical inactivity and smoking using the equation below:

$$PAR=\frac{\mathrm{Prevalence}\times (\mathrm{Relative}\;\mathrm{Risk}-1)}{(P\mathrm{revalence}\times (\mathrm{Relative}\;\mathrm{Risk}-1))+1}$$

This PAR equation was repeated for each sex and age strata (10-year age groups 25–34, 35–44 …… up to 85+). The relative risks used were adjusted for potential confounders.

### Estimating the contributions of medical and surgical treatments

The mortality reduction for each treatment was calculated using the age-specific death rate in each age and sex group multiplied by the relative mortality reduction reported in published meta-analyses, multiplied by the treatment uptake (the proportion of patients receiving that specific treatment).

In cases where a treatment was in use in 1990, the number of DPPs as a result of the therapy used in 1990 was calculated and subtracted from the number of deaths for 2012 to calculate the net benefit. Adjustments were made to account for poly-pharmacy using the Mant and Hicks approach[27]. This approach assumes that for an individual receiving multiple interventions, case fatality reduction is very unlikely to be additive and thus a cumulative relative benefit is estimated. For blood pressure and cholesterol we separated the DPPs from pharmacological versus non-pharmacological (lifestyle) changes by subtracting the DPPs calculated in the treatment arm of the model from the DPPs calculated in the risk factor component. The resulting DPPs were attributed to lifestyle changes.

### Sensitivity analyses

This version of the IMPACT model uses the Excel add-in Ersatz software to perform a probabilistic analysis around the estimates, using the parameter distributions in Appendices C and D. Ersatz allows for repeatedly sampling (1000 iterations performed) random values from the parameter distributions (S7 Table) for the input variables. The 95% uncertainty intervals for the output variable (deaths prevented or postponed) are then calculated. We conducted alternative analyses replacing the GBD risk factor data (smoking, systolic blood pressure, body mass index, diabetes and cholesterol) with data obtained from published articles of national and sub-national population-based surveys conducted in Barbados (S3 Table) for 1990. Finally, we used in a sensitivity analysis GBD risk factor estimates for both 1990 and 2012.

### Ethics

Ethical approval was obtained from the Institutional Review Board of the University of the West Indies; as well as the internal ethics committees of the Queen Elizabeth Hospital and the Ministry of Health (the government agency responsible for management of the public primary care centres). All methods were performed in accordance with the relevant guidelines and regulations of all ethical boards identified. Written informed consent was obtained from participants as required.

## Results

In 1990 the age-standardized CHD mortality rate was 109.5 per 100,000 per year, falling to 55.3 per 100,000 in 2012, a 46.1% decline in CHD deaths over this period (Table 1). This resulted in 139 fewer deaths being observed in 2012 compared with the expected number had the rates remained the same as in 1990. The model explained overall 75% (95% UI 59%, 96%) of deaths prevented or postponed (DPPs). Approximately 56%, 95% UI (46%, 68%) of these deaths were prevented because of the implementation of effective treatment for CHD while the best estimate from the model indicated that decline in risk factors prevented 19% additional deaths 95% UI (6%, 34%).

**Table 1:** CHD mortality rates 1990 and 2012 by sex.

**Male**	1990	2012
**Population (Over 25) (000s)**	69	87
**Observed CHD deaths**	101	81
**Age-standardized rate (00,000)**	133.2	72.3
	
**Expected deaths**	N/A*	144
**Target DPPs**	N/A	63
**% of expected deaths prevented**	N/A	43.8
**Female**	1990	2012
**Population (Over 25) (000s)**	81	99
**Observed CHD deaths**	120	81
**Age-standardized rate (00,000)**	90.6	41.9
**Expected deaths**	N/A	157
**Target DPPs**	N/A	76
**% of expected deaths prevented**	N/A	48.0
**Total**	1990	2012
**Population (Over 25) (000s)**	**149**	**183**
**Observed CHD deaths**	221	162
**Age-standardized rate (00,000)**	109.5	55.3
**Expected deaths**	N/A	301
**Total DPPs**	N/A	139
**% of expected deaths prevented**	N/A	46.1%

*N/A = Not applicable to this time period

### Changes in major CHD risk factors

Age-adjusted risk factor improvements were noted with declines in total cholesterol (14.1% and 12.7% relative decreases in men and women respectively); reduced mean systolic blood pressure in women (1.3%); a reduction in physical inactivity in men(31.1%); and increases in mean fruit and vegetable intake in both sexes (17.9% and 19.4% in men and women respectively) (Table 2). However, the most impactful risk factor increase was the adverse trend in diabetes prevalence where rates increased by 6.5% in men and 41.5% in women (Table 2). Mean BMI also increased in men and women by 5.3 and 6.6% respectively. Smoking prevalence rates in Barbados in 1990 were relatively low (10.5% in men and 1.80% in women) and by 2012 rates were similar 11.1% and 2.3% in men and women respectively.

**Table 2:** Estimated absolute and relative age-adjusted changes in risk factor prevalence/mean levels occurring in Barbados from 1990 to 2012 comparing models 1 and 2.

	Absolute level of risk factor		Change in risk factor^d^		Changes in risk factors–Model 2	
Population risk factor	1990	2012^c^	Absolute risk reduction (ARR)	Relative risk reduction (%)	Absolute risk reduction ARR)	Relative risk reduction (RRR)
**Prevalence of smoking (%)**^**a**^** **						
Men	10.5	11.1	0.6	18.1%	0.003	2.5%
Women	1.8	2.3	0.5	21.7%	0.001	3.5%
**Systolic Blood pressure (mmHg)(anti-hypertensive treatment effects subtracted)**^**a**^						
Men	132.3	132.7	0.4	0.4%	0.1	0.1%
Women	130.7	128.6	-2.1	-1.3%	-1.2	-0.9%
**Total cholesterol (mmol/l) statin effects subtracted**^**a**^						
Men	5.0	4.2	-0.8	-14.1%	-0.3	-4.8%
Women	5.2	4.4	-0.8	-12.7%	-0.2	-3.2%
**Physical inactivity (%) (Base year-2007)**^**b**^						
Men	43.0	31.0	-12.0	-31.1%	-12.0	-31.1%
Women	59.3	67.2	7.9	15.1%	7.9	15.1%
**Body Mass Index **^**a**^						
Men	25.2	26.6	1.4	5.3%	1.5	5.9%
Women	27.8	29.7	1.8	6.6%	1.9	6.8%
**Prevalence of diabetes**^**a**^						
Men	12.4	16.3	3.9	6.5%	0.01	11.4%
Women	13.1	21.6	8.5	41.5%	0.04	35.9%
**Mean Fruit and Vegetables/day (Base year– 2007)**^**b**^						
Men	1.9	2.2	0.3	17.9%	0.3	17.9%
Women	2.0	2.4	0.4	19.4%	0.4	19.4%

a Data for base year obtained from Global Burden of Disease records.

b Data for base year obtained from Barbados STEP Risk Factor Survey 2007.

c Data for 2012 obtained from Health of the Nation Survey in all cases

d Decreases in risk factor levels denoted by a “–” sign.

### Deaths prevented or postponed from medical and surgical treatments

Medical and surgical treatments together prevented/postponed approximately 78 deaths 95% UI(69, 86) in 2012. Improvements in the initial treatments of acute myocardial infarction accounted for the most deaths prevented by an in-hospital treatment modality, 13% (10%, 17%) of the DPPs (Table 3). Antihypertensive treatment in the primary care was also effective, accounting for 9% (7%,11%) of DPPs, while statin treatment accounted for 5% (3%,6%) DPPs. The impact of secondary prevention after AMI was also relatively modest due to the high case-fatality rates following AMI in Barbados[28]- secondary prevention following AMI accounted for 8.6% of DPPs 95% UI (6.7,11.0). The unavailability of Primary PCI in the public sector during the time period and limited availability of coronary artery-bypass grafting resulted in no deaths prevented via secondary prevention after these interventions (S5 & S6 Tables).

**Table 3:** Estimated numbers of deaths from coronary heart disease prevented or postponed by medical and surgical treatments in Barbados in 2012.

	M&H* Net DPPs Best estimate(95% UI)	% of total DPPs Best estimate(95% UI)
**INITIAL TREATMENTS FOR ACUTE MI**	**18.4(15.4,21.7)**	**13.3%(10.4,17.0)**
**UNSTABLE ANGINA**	**12.7(10.1,15.8)**	**9.2%(6.9,12.0)**
**SECONDARY PREVENTION FOLLOWING AMI**	**11.9(9.8,14.5)**	**8.6%(6.7,11.0)**
**SECONDARY PREVENTION FOLLOWING CABG/PCI**	**0.2(0.2,0.3)**	**0.2%(0.1,0.2)**
**ANGINA IN THE COMMUNITY**	**3.8(2.9,4.8)**	**2.8%(2.0,3.7)**
**HEART FAILURE IN THE HOSPITAL**	**6.4(4.8,8.2)**	**4.6%(3.4,6.2)**
**HEART FAILURE IN THE COMMUNITY**	**6.1(4.7,6.8)**	**4.1%(3.2,5.2)**
**STATINS FOR PRIMARY PREVENTION**	**6.2(4.7,8.0)**	**4.5%(3.3,6.1)**
**ANTIHYPERTENSIVE MEDICATION**	**12.1(6.0,18.1)**	**8.8%(4.6,13.2)**

*M&H–Mant and Hicks

### Deaths prevented or postponed from changes in risk factors

Risk factor improvements in total cholesterol levels, systolic blood pressure, fruit and vegetable consumption and physical inactivity accounted for the postponement of 52 deaths, but worsening trends in other risk factors (smoking, diabetes and obesity) were responsible for an additional 26 deaths in 2012, resulting in an overall risk factor effect of 26 DPPs (95% UI 8, 45). Approximately 25% of deaths remained unexplained. Cholesterol declines were the most positively impactful risk factor resulting in 28% (21%, 37%) of DPPs; most of which (22%) were attributed to lifestyle changes.

**Fig 1:**
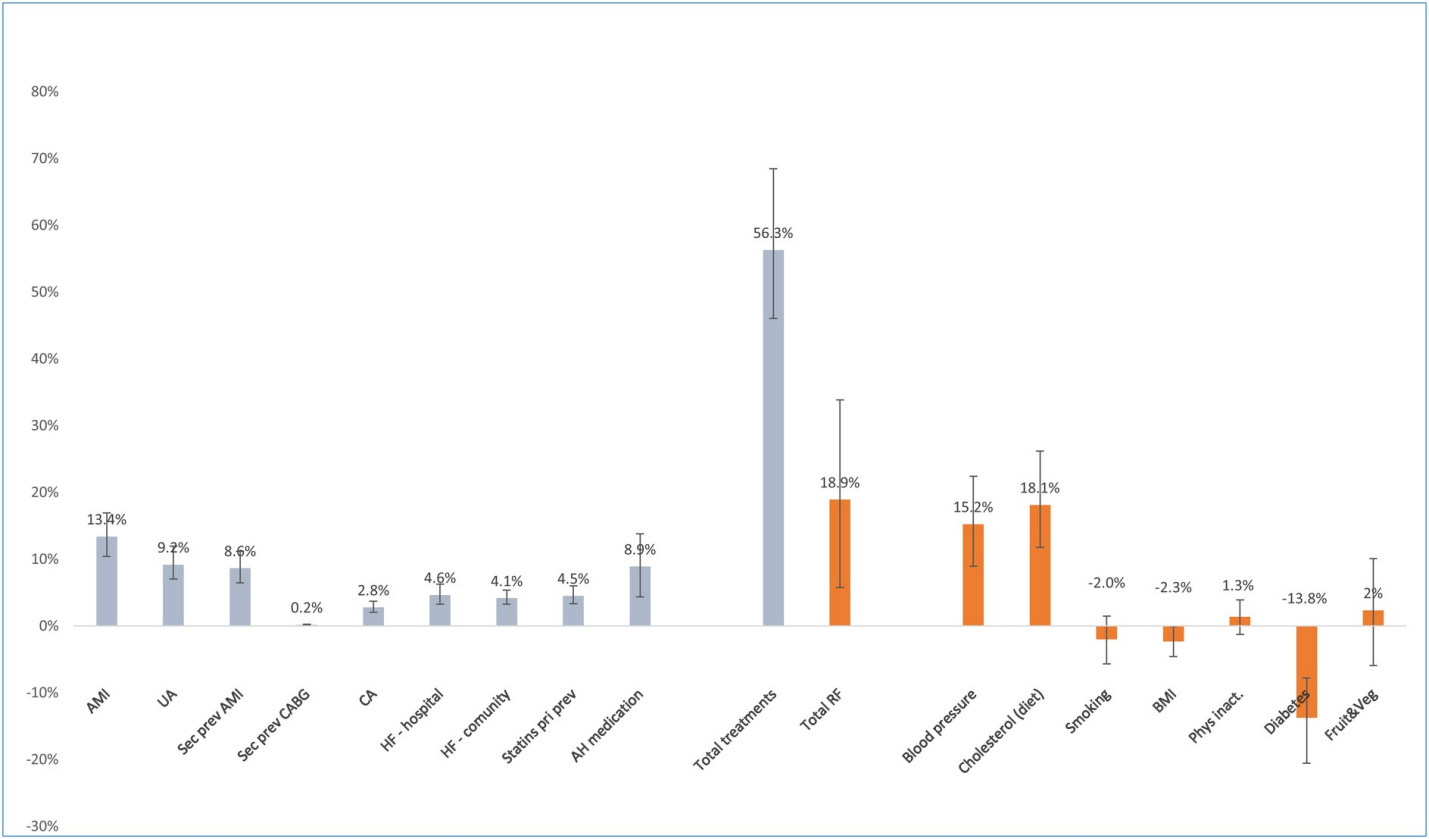
Percentage deaths prevented or postponed for several treatment groups and major cardiovascular risk factors in Barbados from 1990 to 2012.

Fig 1 summarises the DPPs or additional deaths from each treatment and risk factor trend, with the bars representing lower and upper uncertainty intervals generated from the probabilistic analysis. In the overall population systolic blood pressure accounted for 24% of DPPs; 9% as a result of improved coverage of anti-hypertensive therapy (Table 3) and 15% as a result of secular trends.

### Sensitivity analyses

Sensitivity analyses were conducted using Global Burden of Disease risk factor data for both 1990 and 2012. In this alternative model the total model fit was reduced to 49% (34.0%, 66%), with the difference mainly due to varying estimates of the change of systolic blood pressure, total cholesterol and diabetes prevalence. Broadly, the contribution of cholesterol to CHD mortality reduction was reduced (Table 2).

## Discussion

Using the IMPACT model for the small island developing state of Barbados, we found that 56% of the decline in CHD mortality was due to the introduction or improved uptakes of effective treatments while 19% was due to underlying change in risk factors. The highest percentages of DPPs were for treatments given in the first 24 hours for myocardial infarction and for treatment of hypertension and high cholesterol in primary care. Given the small size of the population the uncertainty intervals around our point estimates were wide; the lower and upper uncertainty intervals for the main model fit were 60% and 96% respectively and results must be interpreted accordingly.

### Risk factor changes

The most important risk factor change contributing to the decline in CHD deaths was the high proportion of deaths postponed because of changes in mean cholesterol levels in the population. We estimated that only one third of this reduction was attributed to the introduction of statins. It is possible that dietary changes could explain some of the decline in mean cholesterol levels, but we may also have underestimated the use of statins in our retrospective chart review. Chart reviews often underestimate treatments given since health care providers may have less than stellar documentation practices and handwriting may be difficult for abstractors to decipher despite training. A small study by Sheehy et al suggested that even though energy from fat intake increased from 1961 to 2003 the contribution of saturated fats to total energy decreased over this time period[29]. Most of this decrease appeared to have occurred in the decade of the eighties with no real change noted from 1990 until the end of the study in 2003 [29].

The very low contribution of risk factor reductions to the decline of CHD should be of significant concern to policy makers in Barbados. It demonstrates an opportunity for all of society in a “Health in all Policies[30]” approach to implement population wide programs to change the current tide of rapidly increasing risk factors. Truly upstream approaches must be the priority so that the declines in mean systolic blood pressure or cholesterol or glucose levels will not be driven by medication but rather by lifestyle changes.

There has been some effort made at the local and regional level in the Caribbean to reduce the chronic disease risk factor burden. At the regional level the Port of Spain NCD declaration in 2007 and globally the 2011 UN High Level Meeting represent political leadership and commitment to the reduction of NCD risk, morbidity and mortality. The impact that these declarations have had on risk factor burden is unclear and the initial results of a study to evaluate the outcomes has shown that “NCDs are not given the political priority that is thought to be required to reduce the burden[31]”.

Caribbean countries have reportedly had similar trends in risk factors [32] across the region but varying coronary heart disease mortality outcomes[33], suggesting some variations in the prevention strategies and management of the risk factors and/or outcomes. These variations may be due to a combination of health care system structure and the policy environment. The fact that most of the decline in CHD mortality in Barbados was due to treatment provides an example for SIDs about the advantages of universal access to care and treatment.

### Treatment approaches

Barbados, during the time period of this analysis began to offer fibrinolytic treatment for reperfusion but does not yet provide primary percutaneous intervention (PCI) for acute myocardial infarction routinely. PCI has been associated with lower mortality rates than fibrinolytic therapy but this is true in settings where there is a sufficiently high volume of cardiac events and the staffing required for 24-hour coverage of the catheterization laboratory[34, 35]. Further work is needed to explore the potential impact of introducing PCI versus focusing on increasing uptake of fibrinolytic therapy given the number of STEMI cases seen in this generally low resource setting.

### Model fit

**Fig 2:**
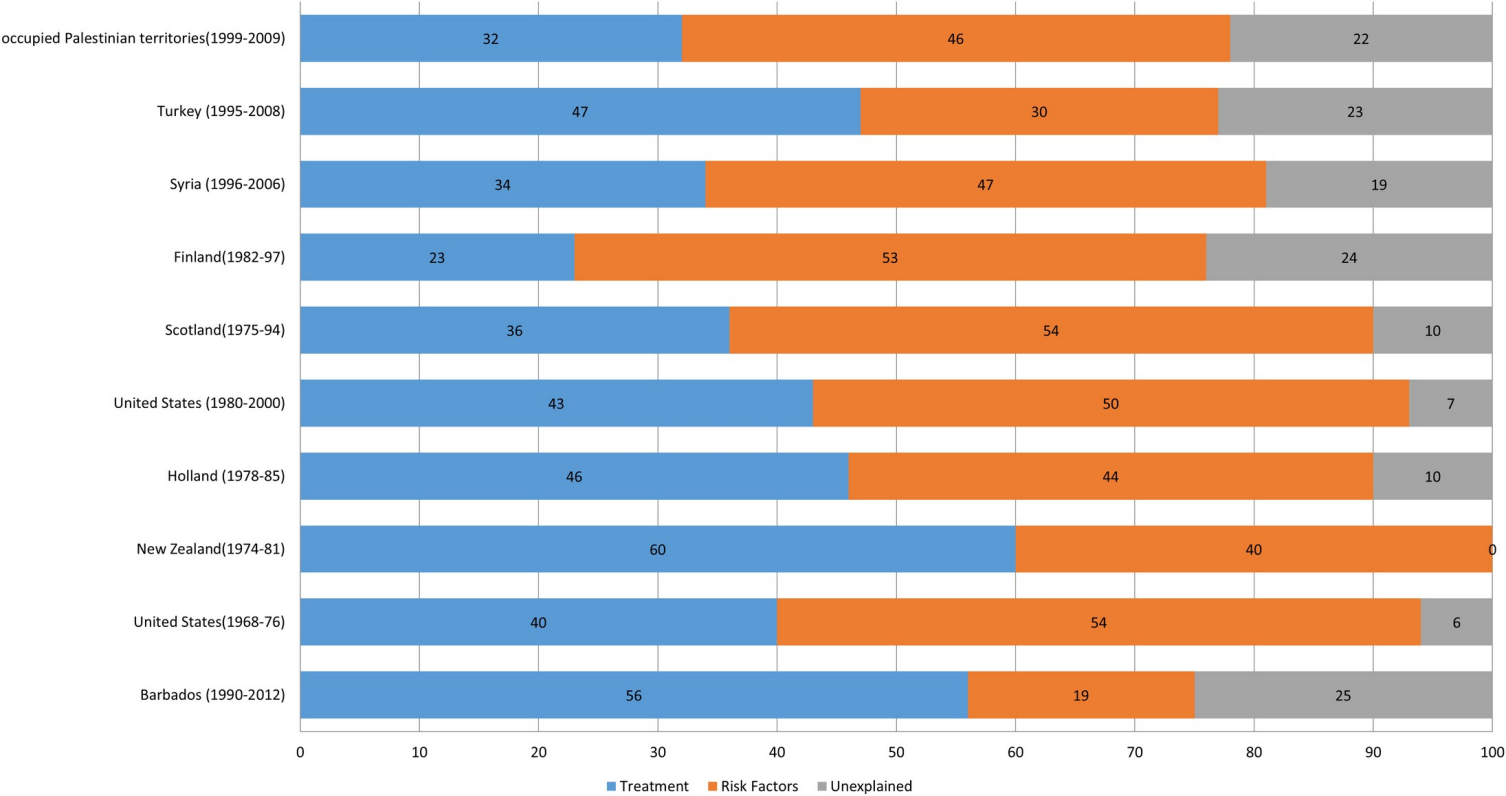
Relative contributions of treatments and risk factors to CHD mortality changes in various countries.

The overall model fit (75%) was similar to Finland, Turkey and Palestine (76%, 77% and 78% respectively (Fig 2), although the uncertainty intervals suggest it could be as much as 96%. In most other countries where the IMPACT model has been used, risk factor changes have made larger contributions to the declines noted in CHD[7, 9, 10]. Smoking was one of the major risk factors whose rates declined dramatically in other countries and thus was a major risk factor contributor to CHD declines[36]. In Barbados, smoking rates were recorded as relatively low in the 1990s. Thus its contribution to the decline in deaths in 2012 was understandably low.

Our use of a qualitative study to inform our assumptions about study estimates in the base year represents one of the strengths of our study, providing contextual information around the progression of myocardial infarction management in the island.

We explored the uncertainty in the data by using rates and mean values estimated from the Global Burden of Disease project for both time periods but found that risk factors explained only a small proportion of the decline noted in CHD mortality. The fact that the relative risks being used in the model were adapted from international sources, may have underestimated the strength of association between diabetes or BMI and antihypertensive use and CHD risk in this Afro-Caribbean population. Individual relative risk associations have been found to be different by ethnicity[37]. We accounted for this difference by ethnicity by using relative risks for diabetes that were specific to an African population[38] but were unable to do this for the other risk factors.

### Limitations

Since our treatment coverage data were taken from patients notes where there may be underreporting of medications prescribed, we may be underestimating the effects of treatments on CHD mortality decline. There was a lack of quality data on fruit and vegetable intake and physical inactivity levels for the earlier time period but given our findings of 1–2% contribution to DPPs it seems likely that the even with the most extreme sensitivity analyses plausible the impact of changes in these risk factors would be small. It is also possible that improvements in CHD mortality coding over time could be resulting in an over-estimate of the decline in CHD mortality in Barbados[39]. Data from the Pan American Health Organization suggests that mortality coding quality has improved in Caribbean countries over the time period being studied[40].

## Conclusion

We found that CHD mortality rates declined by approximately 46.1% in Barbados from 1990 to 2012. Approximately 56% of this decline can be attributed to the introduction and increased uptake of medical treatments, mostly drug therapies used in the first 24 hours after AMI as well as statins and anti-hypertensives prescribed in primary care. It is imperative that future research explores the feasibility and cost-effectiveness of introducing primary PCI as well as increasing uptake of medical treatments (including fibrinolysis) for CHD. Given the significant negative impact of obesity/diabetes on mortality in this analysis, research that explores factors affecting implementation of evidenced-based preventive strategies (e.g. increased physical activity, elimination of dietary trans-fats) to reduce diabetes and obesity is desperately needed. Exploring the reasons for these trends in other SIDs could facilitate quantifying the impact of universal access to care on CHD trends.

## Supporting information

S1 Table Therapies evaluated in IMPACT. (DOCX)

S2 Table Estimated β coefficients from multiple regression analyses. (DOCX)

S3 Table Main Data Sources for Populating the Barbados IMPACT Model. (DOCX)

S4 Table Treatment Utilization Data Sources. (DOCX)

S5 Table Estimated numbers of deaths from coronary heart disease prevented or postponed by medical and surgical treatments in Barbados in 2012. (DOCX)

S6 Table Estimated numbers of deaths from coronary heart disease prevented or postponed by medical and surgical treatments in Barbados in 2012. (DOCX)

S7 Table Uncertainty analysis: parameter distributions, functions and sources. (DOCX)

S1 Appendix Management of Coronary Heart Disease—Interview guide. (DOCX)

S2 Appendix Methodology and results related to documentary analysis and semi-structured interviews. (DOCX)

S3 Appendix Parameter Estimates. (DOCX)

S1 File Health of the Nation Aggregated Data. (XLSX)

S2 File Minimal Dataset for Barbados National Registry for Non-Communicable Disease. (DTA)

S3 File Data dictionary for Barbados National Registry for Non-Communicable Disease. (XLSX)
