# Supplementary material for: Adverse risk factor trends limit gains in coronary heart disease mortality in Barbados: 1990-2012
Source: PLoS One. 2019 Apr 17;14(4):e0215392. doi: 10.1371/journal.pone.0215392 (PMC6469800; doi:10.1371/journal.pone.0215392)
Supplement: S1 Table — (DOCX) [file pone.0215392.s001.docx]

# S1 Table: Therapies evaluated in IMPACT^1^

#

Clinical efficacy of interventions: relative risk reductions obtained from meta-analyses, and randomised clinical trials

| **Treatments** | **Relative risk reduction^†^** | **Comments** | **Source paper: First author (year), notes** |
| --- | --- | --- | --- |
| ***ST elevation myocardial infarction (STEMI)*** | | | |
|  |  |  |  |
| **Thrombolysis** | 31% (95% CI: 14,45) | <55 years: Odds Ratio (OR)=0.692; Relative Risk Reduction (RRR)=30.8% (95% CI: 14,45)  55-64 years: OR=0.736; RRR=26.4% (95% CI: 17,40)  65-74 years: OR=0.752; RRR=24.8% (95% CI: 15,37)  > 75 years: OR=0.844; RRR=15.6% (95% CI: 4,30) | Estess (2002)^2^ |
| **Aspirin** | 23% (95% CI: 15,30) | RRR=23% (95% CI: 15,30): outcome is vascular deaths | ISIS-2 (1988)^3^ |
| **Primary CABG surgery** | 39% (95% CI: 23,52) | OR=0.61 (95% CI: 0.48,0.77); RRR=39% (95% CI: 23,52) 0-5 year mortality | Yusuf (1994)^4^ |
| **Primary PCI** | 30% (95% CI: 15,42) | OR=0.70 (95% CI: 0.58,0.85); RRR=30% (95% CI: 15,42) outcome compares primary angioplasty to thrombolytics | Keeley (2001)^5^ |
| **Beta blockers** | 4% (95% CI: -8,15) | OR=0.96 (95% CI: 0.85,1.08); RRR=4% (95% CI: -8,15) | Freemantle (1999)^6^ |
| **ACE inhibitors** | 7% (95% CI: 2,11) | OR=0.93 (95% CI: 0.89,0.98); RRR=7% (95% CI: 2,11) for 30 day mortality in myocardial infarction | ACE Inhibitor Myocardial Infarction Collaborative Group (1998)^7^ |
| **Clopidogrel** | 3% (95% CI: 1,6) | RRR=3% (95% CI: 1,6) for 30 day mortality in myocardial infarction | Chen (2005)^8^  Sabatine (2005)^9^ |
| **Hospital CPR** | 33% (95% CI: 10,36) | Survival at 24 hours estimated to be 32%, discharge to home at 21%, and 1 year survival to be 15% overall. | Tunstall-Pedoe (1992)^10^  Nadkarni(2010)^11^ |
| ***Non-ST-segment elevation acute coronary syndrome (NSTEACS):*** | | | |
|  |  |  |  |
| **Aspirin alone** | 15% (95% CI: 11,19) | OR=0.85 (95% CI: 0.49,0.95); RRR=15% (95% CI: 11,19). Assume appropriate for patients with NSTE-ACS | Antithrombotic Trialists’ Collaboration (2002)^12^ |
| **Aspirin & heparin** | 33% (95% CI: -2,56) | OR=0.67 (95% CI: 0.48,1.02); RRR=33% (95% CI: -2,56%). The study outcome is composite MI death and non-fatal MI; compares those on aspirin & heparin to aspirin only | Oler (1996)^13^ |
| **Platelet glycoprotein IIB/IIIA inhibitors** | 9% (95% CI: 2,16) | OR=0.91 (95% CI: 0.84,0.98); RRR=9% (95% CI: 2,16). Study looked at acute coronary syndrome without persistent ST elevation. | Boersma (2002)^14^ |
| **Early PCI** | 32% (95% CI: 5,51) | OR=0.68 (95% CI: 0.49,0.95); RRR=32% (95% CI: 5,51) | Fox (2002)^15^ |
| **Primary CABG surgery** | 39% (95% CI: 23,52) | OR=0.61 (95% CI: 0.48,0.77); RRR=39% (95% CI: 23,52) 0-5 year mortality | Yusuf (1994)^4^  Assumed similar as STEMI |
| **Clopidogrel** | 7% (95% CI: 2,11) | RRR=7% (95% CI: 2,11) | Yusuf (2001)^16^ |
| **Beta blockers** | 4% (95% CI: -8,15) | OR=0.96 (95% CI: 0.85,1.08); RRR=4% (95% CI: -8,15) | Freemantle (1999)^6^  Assumed similar as STEMI |
| **ACE inhibitors** | 7% (95% CI: 2,11) | OR=0.93 (95% CI: 0.89,0.98); RRR=7% (95% CI: 2,11) for 30 day mortality in myocardial infarction | ACE Inhibitor Myocardial Infarction Collaborative Group (1998)^7^ |
| ***Secondary prevention post myocardial infarction/revascularisation:*** | | | |
|  |  |  |  |
| **Aspirin** | 15% (95% CI: 11,19) | OR=0.85 (95% CI: 0.49,0.95); RRR=15% (95% CI: 11,19) | Antithrombotic Trialists’ Collaboration (2002)^3^ |
| **Beta blockers** | 23% (95% CI: 15,31) | OR=0.77 (95% CI: 0.69,0.85); RRR=23% (95% CI: 15,31) on page 1734. Odds of death in long term trials | Freemantle (1999)^6^ |
| **ACE inhibitors or Angiotensin-II receptor antagonists** | 20% (95% CI: 13,26) | OR=0.80 (95% CI: 0.74,0.87); RRR=20% (95% CI: 13,26) on page 1577, death up to four years [endpoint of study looking at those with heart failure or LV dysfunction] | Flather (2000)^17^ |
| **Statins** | 24% (95% CI: 10,26) | RRR=24% (95% CI: 10,26)  Intensive statin therapy in acute coronary syndromes. | Pignone (2006)^18^ |
| **Warfarin** | 22% (95% CI: 13,31) | OR=0.78 (95% CI: 0.67,0.90); RRR=22% (95% CI: 10,33) | Anand and Yusuf (1999)^19^ |
| **Rehabilitation** | 26% (95% CI: 10,39) | OR=0.74 (95% CI: 0.61,0.90); RRR=26% (95% CI: 10,39) | Taylor (2004)^20^ |
| ***Chronic stable coronary artery disease:*** | | | |
|  |  |  |  |
| **CABG surgery**  **years 0-5** | 39% (95% CI:23,52) | OR = 0.61 (95% CI: 0.48-0.77), RRR 39% (95% CI: 23,52) 5 year mortality | Yusuf (1994)^4^ |
| **CABG surgery**  **years 6-10** | 32% (95% CI: 2,30) | OR = 0.83 (95% CI: 0.70-0.98), RRR 17% (95% CI: 2,30) on page 565, 10 year mortality.  OR = 0.68 (95% CI: 0.56-0.83), RRR 32% (95% CI: 17,44) on page 565, 7 year mortality | Yusuf (1994)^4^ |
| **Angioplasty** | No effect |  | Boden (2007) ^21^ |
| **Aspirin** | 15% (95% CI: 11,19) | OR=0.85 (95% CI: 0.49-0.95); RRR=15% (95% CI: 11,19). Outcome is vascular and nonvascular deaths. | Antithrombotic Trialists’ Collaboration (2002)^12^ |
| **Statins** | 23% (95% CI: 10,26) | RRR=23% (95% CI 10,26)  Standard dose statin therapy in coronary artery disease. | Wilt (2004)^22^ |
| **ACE inhibitors/ARB** | 17% (95% CI: 6,28) | RRR=17% (95% CI 6,28) | Al-Mallah (2006)^23^ |
| ***Heart failure in patients requiring hospitalisation or in the community:*** | | | |
|  |  |  |  |
| **ACE inhibitors** | 20% (95% CI: 13,26) | OR=0.80 (95% CI: 0.74,0.87); RRR=20% (95% CI: 13,26) on page 1577 [death up to four years was study endpoint for those with heart failure or LV dysfunction] | Flather (2000)^17^ |
| **Beta blockers** | 35% (95% CI: 26,43) | OR=0.65 (95% CI: 0.57,0.74); RRR=35% (95% CI: 26,43): all cause mortality | Shibata (2001)^24^ |
| **Spironolactone** | 30% (95% CI: 18,41)  31% (95% CI: 18,42) | OR=0.70 (95% CI: 0.59,0.82); RRR=30% (95% CI: 18,41) in those that had at least one cardiac related hospitalisation.  OR=0.69 (95% CI: 0.58,0.82); RRR=31% (95% CI: 18,42) in entire study population consisting of those with community heart failure, page 711. | Pitt (1999)^25^ |
| **Aspirin** | 15% (95% CI: 11,19) | OR=0.85 (95% CI: 0.49,0.95); RRR=15% (95% CI: 11,19). Outcome is vascular and nonvascular deaths. | Antithrombotic Trialists’ Collaboration (2002)^12^ |
| **Statins** | No effect |  | Kjekshus (2007)^26^  Tavazzi (2008)^27^ |
| ***Primary prevention therapies*** | | | |
|  |  |  |  |
| **Treatments for high blood pressure** | 13% (95% CI: 6,19) | OR=0.87 (95% CI: 0.81,0.94); RRR=13% (95% CI: 6,19) in those with high blood pressure without disease at entry. [RRR=29% (95% CI: 17,37) those with average blood pressure and CHD, treated with ACE inhibitors] | Law (2003)^28^ |
| **Statins** | 35% (95% CI: 11,52) | OR=0.65 (95% CI: 0.48,0.89); RRR=35% (95% CI: 11,52) for CHD mortality (only trials using statins) | Pignone (2000)^18^ |

^†^Relative risk reduction (RRR) calculated as 1 – odds ratio

Adapted from expanded IMPACT methods found in

Bajekal M, Scholes S, Love H, Hawkins N, O'Flaherty M, Raine R, et al. (2012) Analysing Recent Socioeconomic Trends in Coronary Heart Disease Mortality in England, 2000–2007: A Population Modelling Study. PLoS Med 9(6): e1001237. <https://doi.org/10.1371/journal.pmed.1001237>

**References**

1. Bajekal M, Scholes S, Love H, et al. Analysing recent socioeconomic trends in coronary heart disease mortality in England, 2000-2007: a population modelling study. *PLoS Med.* 2012;9(6):e1001237.

2. Estess JM, Topol EJ. Fibrinolytic treatment for elderly patients with acute myocardial infarction. *Heart.* 2002;87(4):308-311.

3. Baigent C, Collins R, Appleby P, Parish S, Sleight P, Peto R. ISIS-2: 10 year survival among patients with suspected acute myocardial infarction in randomised comparison of intravenous streptokinase, oral aspirin, both, or neither. The ISIS-2 (Second International Study of Infarct Survival) Collaborative Group. *Bmj.* 1998;316(7141):1337-1343.

4. Yusuf S, Zucker D, Peduzzi P, et al. Effect of coronary artery bypass graft surgery on survival: overview of 10-year results from randomised trials by the Coronary Artery Bypass Graft Surgery Trialists Collaboration. *Lancet.* 1994;344(8922):563-570.

5. Keeley EC, Velez CA, O'Neill WW, Safian RD. Long-term clinical outcome and predictors of major adverse cardiac events after percutaneous interventions on saphenous vein grafts. *Journal of the American College of Cardiology.* 2001;38(3):659-665.

6. Freemantle N, Cleland J, Young P, Mason J, Harrison J. beta Blockade after myocardial infarction: systematic review and meta regression analysis. *BMJ.* 1999;318(7200):1730-1737.

7. Indications for ACE inhibitors in the early treatment of acute myocardial infarction: systematic overview of individual data from 100,000 patients in randomized trials. ACE Inhibitor Myocardial Infarction Collaborative Group. *Circulation.* 1998;97(22):2202-2212.

8. Chen ZM, Jiang LX, Chen YP, et al. Addition of clopidogrel to aspirin in 45,852 patients with acute myocardial infarction: randomised placebo-controlled trial. *Lancet.* 2005;366(9497):1607-1621.

9. Sabatine MS, Cannon CP, Gibson CM, et al. Addition of clopidogrel to aspirin and fibrinolytic therapy for myocardial infarction with ST-segment elevation. *The New England journal of medicine.* 2005;352(12):1179-1189.

10. Tunstall-Pedoe H, Bailey L, Chamberlain DA, Marsden AK, Ward ME, Zideman DA. Survey of 3765 cardiopulmonary resuscitations in British hospitals (the BRESUS Study): methods and overall results. *Bmj.* 1992;304(6838):1347-1351.

11. Nadkarni VM, Nolan JP, Billi JE, et al. Part 2: International collaboration in resuscitation science: 2010 International Consensus on Cardiopulmonary Resuscitation and Emergency Cardiovascular Care Science With Treatment Recommendations. *Circulation.* 2010;122(16 Suppl 2):S276-282.

12. Antithrombotic Trialists C. Collaborative meta-analysis of randomised trials of antiplatelet therapy for prevention of death, myocardial infarction, and stroke in high risk patients. *Bmj.* 2002;324(7329):71-86.

13. Oler A, Whooley MA, Oler J, Grady D. Adding heparin to aspirin reduces the incidence of myocardial infarction and death in patients with unstable angina. A meta-analysis. *Jama.* 1996;276(10):811-815.

14. Boersma E, Harrington RA, Moliterno DJ, et al. Platelet glycoprotein IIb/IIIa inhibitors in acute coronary syndromes: a meta-analysis of all major randomised clinical trials. *Lancet.* 2002;359(9302):189-198.

15. Fox KA, Poole-Wilson PA, Henderson RA, et al. Interventional versus conservative treatment for patients with unstable angina or non-ST-elevation myocardial infarction: the British Heart Foundation RITA 3 randomised trial. Randomized Intervention Trial of unstable Angina. *Lancet.* 2002;360(9335):743-751.

16. Yusuf S, Zhao F, Mehta SR, et al. Effects of clopidogrel in addition to aspirin in patients with acute coronary syndromes without ST-segment elevation. *The New England journal of medicine.* 2001;345(7):494-502.

17. Flather MD, Yusuf S, Kober L, et al. Long-term ACE-inhibitor therapy in patients with heart failure or left-ventricular dysfunction: a systematic overview of data from individual patients. ACE-Inhibitor Myocardial Infarction Collaborative Group. *Lancet.* 2000;355(9215):1575-1581.

18. Pignone M, Earnshaw S, Tice JA, Pletcher MJ. Aspirin, statins, or both drugs for the primary prevention of coronary heart disease events in men: a cost-utility analysis. *Annals of internal medicine.* 2006;144(5):326-336.

19. Anand SS, Yusuf S. Oral anticoagulant therapy in patients with coronary artery disease: a meta-analysis. *JAMA.* 1999;282(21):2058-2067.

20. Taylor RS, Brown A, Ebrahim S, et al. Exercise-based rehabilitation for patients with coronary heart disease: systematic review and meta-analysis of randomized controlled trials. *The American journal of medicine.* 2004;116(10):682-692.

21. Boden WE, O'Rourke RA, Teo KK, et al. Optimal medical therapy with or without PCI for stable coronary disease. *The New England journal of medicine.* 2007;356(15):1503-1516.

22. Wilt TJ, Bloomfield HE, MacDonald R, et al. Effectiveness of statin therapy in adults with coronary heart disease. *Archives of internal medicine.* 2004;164(13):1427-1436.

23. Al-Mallah MH, Tleyjeh IM, Abdel-Latif AA, Weaver WD. Angiotensin-converting enzyme inhibitors in coronary artery disease and preserved left ventricular systolic function: a systematic review and meta-analysis of randomized controlled trials. *Journal of the American College of Cardiology.* 2006;47(8):1576-1583.

24. Shibata MC, Flather MD, Wang D. Systematic review of the impact of beta blockers on mortality and hospital admissions in heart failure. *European journal of heart failure.* 2001;3(3):351-357.

25. Pitt B, Zannad F, Remme WJ, et al. The effect of spironolactone on morbidity and mortality in patients with severe heart failure. Randomized Aldactone Evaluation Study Investigators. *The New England journal of medicine.* 1999;341(10):709-717.

26. Kjekshus J, Pedersen TR, Olsson AG, Faergeman O, Pyorala K. The effects of simvastatin on the incidence of heart failure in patients with coronary heart disease. *J Card Fail.* 1997;3(4):249-254.

27. Tavazzi L, Maggioni AP, Marchioli R, et al. Effect of rosuvastatin in patients with chronic heart failure (the GISSI-HF trial): a randomised, double-blind, placebo-controlled trial. *Lancet.* 2008;372(9645):1231-1239.

28. Law MR, Morris JK, Wald NJ. Use of blood pressure lowering drugs in the prevention of cardiovascular disease: meta-analysis of 147 randomised trials in the context of expectations from prospective epidemiological studies. *Bmj.* 2009;338:b1665.
