# Supplementary material for: Adverse risk factor trends limit gains in coronary heart disease mortality in Barbados: 1990-2012
Source: PLoS One. 2019 Apr 17;14(4):e0215392. doi: 10.1371/journal.pone.0215392 (PMC6469800; doi:10.1371/journal.pone.0215392)
Supplement: S2 Table — (DOCX) [file pone.0215392.s002.docx]

# S2 Table: Estimated β coefficients from multiple regression analyses

Estimated β coefficients from multiple regression analyses for the relationship between absolute changes in population mean risk factors and percentage changes in coronary heart disease mortality for men and women, stratified by age.

| **Systolic blood pressure** | | **Age group (years)** | | | | |
| --- | --- | --- | --- | --- | --- | --- |
|  | | **25-44** | **45-54** | **55-64** | **65-74** | **75+** |
|  | |  |  |  |  |  |
| **Men** (hazard ratio per 20 mmHg) | | 0.49 | 0.49 | 0.52 | 0.58 | 0.65 |
| Men (log hazard ratio per 1 mmHg) | | **-0.036** | **-0.035** | **-0.032** | **-0.027** | **-0.021** |
|  | |  |  |  |  |  |
| *Minimum* | | *-0.029* | *-0.028* | *-0.026* | *-0.022* | *-0.017* |
| *Maximum* | | *-0.043* | *-0.042* | *-0.039* | *-0.032* | *-0.025* |
|  | |  |  |  |  |  |
|  | |  |  |  |  |  |
| **Women** (hazard ratio per 20 mmHg) | | 0.40 | 0.40 | 0.49 | 0.52 | 0.59 |
| Women (log hazard ratio per 1 mmHg) | | **-0.046** | **-0.046** | **-0.035** | **-0.032** | **-0.026** |
| *Minimum* | | *-0.037* | *-0.037* | *-0.028* | *-0.026* | *-0.021* |
| *Maximum* | | *-0.055* | *-0.055* | *-0.042* | *-0.039* | *-0.031* |
|  | |  |  |  |  |  |
| Source: Prospective studies collaborative meta-analysis, Lancet 2002^1^ | | | | | | |
| Units: Percentage change in CHD mortality per 20 mmHg change in systolic blood pressure | | | | | | |
|  |  | | | | | |

| **Cholesterol** | **Age groups (years)** | | | | | | |
| --- | --- | --- | --- | --- | --- | --- | --- |
|  | **25-44** | | **45-54** | **55-64** | **65-74** | **75-84** | **85+** |
| **Mortality reduction per 1 mmol/l** | | | | | | | |
| Men | 0.55 | | 0.53 | 0.36 | 0.21 | 0.21 | 0.21 |
| Women | 0.57 | | 0.52 | 0.35 | 0.23 | 0.23 | 0.23 |
| **Log coefficient** | | | | | | | |
| **Men** | **-0.799** | | **-0.755** | **-0.446** | **-0.236** | **-0.117** | **-0.083** |
| *Minimum* | *-0.639* | | *-0.604* | *-0.357* | *-0.189* | *-0.093* | *-0.067* |
| *Maximum* | *-0.958* | | *-0.906* | *-0.536* | *-0.283* | *-0.140* | *-0.100* |
|  |  | |  |  |  |  |  |
| **Women** | **-0.844** | | **-0.734** | **-0.431** | **-0.261** | **-0.174** | **-0.051** |
| *Minimum* | *-0.675* | | *-0.587* | *-0.345* | *-0.209* | *-0.139* | *-0.041* |
| *Maximum* | *-1.013* | | *-0.881* | *-0.517* | *-0.314* | *-0.209* | *-0.062* |
| Source: Prospective studies collaborative meta-analysis, Lancet 2007^2^ | | | | | | | |
| Units: | | Percentage change in CHD mortality per 1 mmol/l change in total cholesterol | | | | | |

| **Body Mass Index (BMI)** | | **Age groups (years)** | | | | |
| --- | --- | --- | --- | --- | --- | --- |
|  | | **<44** | **45-59** | **60-69** | **70-79** | **80+** |
| *James et.al (2004):* | |  |  |  |  |  |
| Hazard ratio | | 0.89 | 0.91 | 0.95 | 0.96 | 0.97 |
| Risk reduction† per 1 kg/m^2^ | | 0.11 | 0.09 | 0.05 | 0.04 | 0.03 |
| Age gradient (45-59 as reference) | | 1.22 | **1.00** | 0.56 | 0.44 | 0.33 |
| *Bogers (2006):*  Relative risks, CHD deaths per 5 BMI units (kg/m^2^) | |  | **1.16** |  |  |  |
| Relative risks per 1 kg/m^2^ applying age gradients from James et.al | | 1.04 | 1.03 | 1.02 | 1.01 | 1.01 |
| **Log coefficients** | | **0.0363** | **0.0297** | **0.0165** | **0.0132** | **0.0099** |
| *Minimum* | | *0.0255* | *0.0209* | *0.0116* | *0.0093* | *0.0070* |
| *Maximum* | | *0.0466* | *0.0381* | *0.0212* | *0.0169* | *0.0127* |
| Source: Bogers et al (2007)^3^, James et al (2004)^4^ | | | | | | |
| Units: | Percentage change in CHD mortality per 1 kg/m^2^ change in BMI | | | | | |

^†^ Risk reduction = 1 – hazard ratio

**References**

1. Lewington S, Clarke R, Qizilbash N, Peto R, Collins R, Prospective Studies C. Age-specific relevance of usual blood pressure to vascular mortality: a meta-analysis of individual data for one million adults in 61 prospective studies. *Lancet.* 2002;360(9349):1903-1913.

2. Prospective Studies C, Lewington S, Whitlock G, et al. Blood cholesterol and vascular mortality by age, sex, and blood pressure: a meta-analysis of individual data from 61 prospective studies with 55,000 vascular deaths. *Lancet.* 2007;370(9602):1829-1839.

3. Bogers RP, Bemelmans WJ, Hoogenveen RT, et al. Association of overweight with increased risk of coronary heart disease partly independent of blood pressure and cholesterol levels: a meta-analysis of 21 cohort studies including more than 300 000 persons. *Archives of internal medicine.* 2007;167(16):1720-1728.

4. James PT, Rigby N, Leach R, International Obesity Task F. The obesity epidemic, metabolic syndrome and future prevention strategies. *European journal of cardiovascular prevention and rehabilitation : official journal of the European Society of Cardiology, Working Groups on Epidemiology & Prevention and Cardiac Rehabilitation and Exercise Physiology.* 2004;11(1):3-8.
