# Supplementary material for: Adverse risk factor trends limit gains in coronary heart disease mortality in Barbados: 1990-2012
Source: PLoS One. 2019 Apr 17;14(4):e0215392. doi: 10.1371/journal.pone.0215392 (PMC6469800; doi:10.1371/journal.pone.0215392)
Supplement: S3 Table — (DOCX) [file pone.0215392.s003.docx]

# S3 Table: Main Data Sources for Populating the Barbados IMPACT Model

| **Information required** | **Source: 1990** | **Source: 2012** |
| --- | --- | --- |
| Population Statistics | Barbados Statistical Service | Barbados Statistical Service |
| Deaths by Age and Sex  Number | WHO Mortality database | WHO Mortality database |
| **Number of patients admitted yearly** |  |  |
| Acute Myocardial Infarction (AMI) | Chief Medical Officer(CMO) report for 1991 for AMI | Barbados National Registry for Acute Myocardial Infarction and Stroke |
| Acute Angina | Chief Medical Officer’s Report | Chief Medical Officer’s Report |
| Heart failure | Chief Medical Officer’s Report | Chief Medical Officer’s Report |
| **Number of patients in following treatment categories** | 1990 |  |
| Coronary Artery By-pass Grafting (CABG) | Expert Opinion | Hospital Surgery Log Sheet |
| Percutaneous Coronary Intervention (PCI) | Expert opinion | Expert opinion |
| Post-Myocardial Infarction | CMO report | Barbados National Registry |
| Chronic angina in Community | Assumed to be half of final year | Health of the Nation Survey |
| Heart failure in community | Assumed to be half of final year | Health of the Nation Survey |
| Hypertension (primary prevention in hypertensives) | Assumed to be half of final year | Health of the Nation Survey |
| Hyper-lipidemia (primary prevention in cholesterol to prevent IHD) | Assumed to be half of final year | Health of the Nation Survey |
| **Population Risk Factor Prevalence** | **1990** | **2012** |
| Current cigarette smoking | Barbados Risk Factor Survey (1992) Unpublished study, Government report | Health of the Nation Survey |
| Systolic Blood Pressure | International Study of Hypertension in Blacks (1991-4) | Health of the Nation Survey |
| Total cholesterol | St. James Study(Trinidad) | Health of the Nation Survey |
| Physical inactivity | St. James Study(Trinidad) | Health of the Nation Survey |
| Body Mass Index | International Study of Hypertension in Blacks (1991-4) | Health of the Nation Survey |
| Diabetes | Sub-national study by Foster et al | Health of the Nation Survey |
| Fruits and vegetables | National Risk Factor STEPS survey(2007) | Health of the Nation Survey |
