# Supplementary material for: Adverse risk factor trends limit gains in coronary heart disease mortality in Barbados: 1990-2012
Source: PLoS One. 2019 Apr 17;14(4):e0215392. doi: 10.1371/journal.pone.0215392 (PMC6469800; doi:10.1371/journal.pone.0215392)
Supplement: S4 Table — (DOCX) [file pone.0215392.s004.docx]

# S4 Table: Treatment Utilization Data Sources

| **Acute myocardial infarction** | **1990 Source** | **2012 Source** |
| --- | --- | --- |
| Thrombolysis | Qualitative study/Expert opinion | Barbados National Registry for Chronic Non-communicable Diseases |
| Primary PCI | Qualitative study/Expert opinion | Barbados National Registry for Chronic Non-communicable Diseases |
| Aspirin | Assumption: 25% of Final year | Barbados National Registry for Chronic Non-communicable Diseases |
| Beta Blockers | Assumption: 25% of final year | Barbados National Registry for Chronic Non-communicable Diseases |
| ACE Inhibitor | Qualitative study/Expert opinion | Barbados National Registry for Chronic Non-communicable Diseases |
| Primary CABG | Qualitative study/Expert opinion | Barbados National Registry for Chronic Non-communicable Diseases |
| Community CPR | Qualitative study/Expert opinion | Barbados National Registry for Chronic Non-communicable Diseases |
| Hospital CPR | Qualitative study/Expert opinion | Barbados National Registry for Chronic Non-communicable Diseases |
| Rehabilitation | Qualitative study/Expert opinion | Barbados National Registry for Chronic Non-communicable Diseases |
| **Unstable Angina (DG2)** |  |  |
| Aspirin alone | Assumption: 50% of final year (2012) | Retrospective Chart Review |
| Aspirin and heparin | Assumption: 50% of final year (2012) | Retrospective Chart Review |
| Platelet glycoprotein IIB/IIIA inhibitors | Qualitative study/Expert opinion | Retrospective Chart Review |
| PCI | Qualitative study/Expert opinion | Retrospective Chart Review |
| CABG surgery | Qualitative study/Expert opinion | Retrospective Chart Review |
| **2^nd^ Prevention post AMI** | **1990 Source** | **2012 Source** |
| Aspirin | Assumption: 50% of final year (2012) | Barbados National Registry for Chronic Non-communicable Diseases |
| Beta Blockers | Assumption: 50% of final year (2012) | Barbados National Registry for Chronic Non-communicable Diseases |
| ACE inhibitors | Qualitative study/Documentary analysis | Barbados National Registry for Chronic Non-communicable Diseases |
| Statins | Qualitative study/Documentary analysis | Barbados National Registry for Chronic Non-communicable Diseases |
| Warfarin | Assumption: 50% of final year (2009) | Barbados National Registry for Chronic Non-communicable Diseases |
| Rehabilitation | Qualitative study/Expert opinion | Barbados National Registry for Chronic Non-communicable Diseases |
| **2'prevention following CABG/PTCA** |  |  |
| Statins | Qualitative study/Expert opinion | Retrospective Chart Review |
| Aspirin | Qualitative study/Expert opinion | Retrospective Chart Review |
| ACE inhibitors | Qualitative study/Expert opinion | Retrospective Chart Review |
| Beta-blockers | Qualitative study/Expert opinion | Retrospective Chart Review |
| Warfarin | Qualitative study/Expert opinion | Retrospective Chart Review |
| Rehabilitation | Qualitative study/Expert opinion | Retrospective Chart Review |
| **Chronic angina** |  |  |
| CABG surgery | Qualitative study/Expert opinion | Retrospective Chart Review |
| Angioplasty | Qualitative study/Documentary analysis | Retrospective Chart Review |
| Aspirin | Assumption: 50% of final year | Retrospective Chart Review |
| Statins | Qualitative study/Documentary Analysis | Retrospective Chart Review |
| **Hospital heart failure** |  |  |
| ACE inhibitors | Qualitative study/Documentary Analysis | Retrospective Chart Review |
| Beta blockers | Assumption: 50% of final year | Retrospective Chart Review |
| Spironolactone | Assumption: 50% of final year | Retrospective Chart Review |
| Aspirin | Assumption: 50% of final year | Retrospective Chart Review |
| Statins | Qualitative study/Documentary Analysis | Retrospective Chart Review |
| **Heart failure in the community** |  |  |
| Statins | Qualitative study/Documentary Analysis | Retrospective Chart Review |
| Aspirin | Assumption: 50% of final year | Retrospective Chart Review |
| ACE inhibitors | Qualitative study/Documentary Analysis | Retrospective Chart Review |
| Beta-blockers | Assumption: 50% of final year | Retrospective Chart Review |
| Spironolactone | Assumption: 50% of final year | Retrospective Chart Review |
| **Primary prevention hypertension** |  |  |
| Treated % of total population | Assumption: 50% of final year | Health of the Nation Survey |
| **Primary prevention hyperlipidemia** |  |  |
| Treated % |  |  |
| Statins | Qualitative study/Documentary analysis | Health of the Nation Survey |
