# Supplementary material for: Adverse risk factor trends limit gains in coronary heart disease mortality in Barbados: 1990-2012
Source: PLoS One. 2019 Apr 17;14(4):e0215392. doi: 10.1371/journal.pone.0215392 (PMC6469800; doi:10.1371/journal.pone.0215392)
Supplement: S5 Table — (DOCX) [file pone.0215392.s005.docx]

# S5 Table: Estimated numbers of deaths from coronary heart disease prevented or postponed by medical and surgical treatments in Barbados in 2012

| **INITIAL TREATMENTS FOR ACUTE MI** | **Treatment uptake(2012)** | **M&H Net DPPs**  **Best estimate(95% UI)** | **% of total DPPs**  **Best estimate(95% UI)** |
| --- | --- | --- | --- |
| **Aspirin** | 0.81 | 7.2(5.7,9.0) | 5.2%(4.0,6.9) |
| **ACE inhibitor/ARBs** | 0.37 | 2.0(1.6,2.4) | 1.4%(1.1,1.8) |
| **Beta blockers** | 0.48 | 0.7(0.5,1.0) | 0.5%(0.4,0.7) |
| **CABG** | 0.00 | 0.0(0.0,0.0) | 0.0%(0.0,0.0) |
| **PCI (STEMI)** | 0.00 | 0.0(0.0,0.0) | 0.0%(0.0,0.0) |
| **Rehabilitation** | 0.09 | 0.5(-0.1,1.1) | 0.4%(-0.1,0.8) |
| **Community CPR** | 0.01 | 0.0(0.0,0.1) | 0.0%(0.0,0.0) |
| **Hospital CPR** | 0.08 | 0.1(0.0,0.1) | 0.0%(0.0,0.0) |
| **Thrombolysis** | 0.34 | 6.2(4.9,7.6) | 4.5%(3.4,5.9) |
| **PCI (NSTEMI)** | 0.00 | 0.0(0.0,0.0) | 0.0%(0.0,0.0) |
| **Clopidogrel** | 0.74 | 1.7(1.4,2.0) | 1.2%(1.0,1.6) |
| **Total** |  | **18.4(15.4,21.7)** | **13.3%(10.4,17.0)** |
| **UNSTABLE ANGINA** | | | |
| **Aspirin** | 0.87 | 4.5(3.5,5.6) | 3.3%(2.4,4.4) |
| **PG IIA/IIIB** | 0.35 | 2.0(1.5,2.8) | 1.5%(1.0,2.1) |
| **CABG** | 0.12 | 2.9(1.5,4.5) | 2.1%(1.1,3.4) |
| **PCI (STEMI)** | 0.00 | 0.0(0.0,0.0) | 0.0%(0.0,0.0) |
| **Clopidogrel** | 0.74 | 3.3(2.7,3.9) | 2.4%(1.8,3.0) |
| **Total** |  | **12.7(10.1,15.8)** | **9.2%(6.9,12.0)** |
| **SECONDARY PREVENTION FOLLOWING AMI** | | | |
| **Statins** | 0.51 | 4.1(3.2,5.1) | 3.0%(2.2,3.9) |
| **Aspirin** | 0.67 | 0.9(0.2,1.7) | 0.6%(0.2,1.2) |
| **ACE inhibitors/ARBs** | 0.50 | 3.5(2.7,4.4) | 2.5%(1.9,3.2) |
| **Beta blockers** | 0.59 | 2.4(1.4,3.4) | 1.7%(1.0,2.6) |
| **Rehabilitation** | 0.10 | 0.8(0.5,1.2) | 0.6%(0.4,0.9) |
| **Total** |  | **11.9(9.8,14.5)** | **8.6%(6.7,11.0)** |
| **SECONDARY PREVENTION FOLLOWING CABG/PCI** | | | |
| **Total** |  | **0.2(0.2,0.3)** | **0.2%(0.1,0.2)** |
