# Supplementary material for: Adverse risk factor trends limit gains in coronary heart disease mortality in Barbados: 1990-2012
Source: PLoS One. 2019 Apr 17;14(4):e0215392. doi: 10.1371/journal.pone.0215392 (PMC6469800; doi:10.1371/journal.pone.0215392)
Supplement: S6 Table — (DOCX) [file pone.0215392.s006.docx]

# S6 Table: Estimated numbers of deaths from coronary heart disease prevented or postponed by medical and surgical treatments in Barbados in 2012

| **ANGINA IN THE COMMUNITY** | **Treatment Uptake(2012)** | **M&H Net DPPs** | **% of total DPPs** |
| --- | --- | --- | --- |
| **Statins** | 0.57 | 3.7(2.9,4.4) | 2.6%(2.0,3.4) |
| **Aspirin** | 0.73 | 0.0(-0.5,0.5) | 0.0%(-0.3,0.4) |
| **CABG** | 0.01 | 0.2(0.2,0.6) | 0.1%(0.0,0.5) |
| **PCI (STEMI)** | 0.00 | 0.0(0.0,0.0) | 0.0%(0.0,0.0) |
| **Total** |  | **3.8(2.9,4.8)** | **2.8%(2.0,3.7)** |
| **HEART FAILURE IN THE HOSPITAL** | | | |
| **Aspirin** | 0.58 | 0.6(0.2,1.0) | 0.4%(0.1,0.7) |
| **ACE inhibitors/ARBs** | 0.73 | 1.7(1.2,2.2) | 1.2%(0.9,1.7) |
| **Beta blockers** | 0.82 | 2.5(1.7,3.4) | 1.8%(1.2,2.5) |
| **Spironolactone** | 0.62 | 1.7(1.1,2.3) | 1.2%(0.8,1.8) |
| **Total** |  | **6.4(4.8,8.2)** | **4.6%(3.4,6.2)** |
| **HEART FAILURE IN THE COMMUNITY** | | | |
| **Aspirin** | 0.59 | 0.4(0.1,0.7) | 0.3%(0.0,0.5) |
| **ACE inhibitors/ARBs** | 0.49 | 1.2(0.9,1.6) | 0.9%(0.6,1.2) |
| **Beta blockers** | 0.57 | 2.8(2.1,3.5) | 2.0%(1.4,2.7) |
| **Spironolactone** | 0.30 | 1.3(0.8,1.8) | 0.9%(0.6,1.4) |
| **Total** |  | **6.1(4.7,6.8)** | **4.1%(3.2,5.2)** |
| **STATINS FOR PRIMARY PREVENTION** | | | |
| **Total** | 0.35 | 6.2(4.7,8.0) | **4.5%(3.3,6.1)** |
| **ANTIHYPERTENSIVE MEDICATION** | | | |
| **Total** | 0.42 | **12.1(6.0,18.1)** | **8.8%(4.6,13.2)** |
