# Supplementary material for: Adverse risk factor trends limit gains in coronary heart disease mortality in Barbados: 1990-2012
Source: PLoS One. 2019 Apr 17;14(4):e0215392. doi: 10.1371/journal.pone.0215392 (PMC6469800; doi:10.1371/journal.pone.0215392)
Supplement: S7 Table — (DOCX) [file pone.0215392.s007.docx]

# S7 Table: Uncertainty analysis: parameter distributions, functions and sources

We implemented stochastic uncertainty analysis in Excel using Ersatz (version 1.35 available at <http://www.epigear.com>), an add-in that allows probabilistic sensitivity analysis in Excel. Ersatz allows repeated random draws from specified distributions for input variables that are used to recalculate the model. It then calculates the 95% uncertainty intervals from the realised values of the output variable (deaths prevented or postponed). Input variables taken from external sources (e.g. case fatality rates, beta coefficients and relative risk reductions) were randomly drawn from specified distributions.

The table below records the type of distribution and associated functions for each of the input variables in the IMPACT BARBADOS model.

| **Input parameters** | ***Type of distribution and functions (Mean, Standard error)*** | Source |
| --- | --- | --- |
| **Population** | | |
| Population counts and CHD deaths stratified by age, sex | - Population counts (no error) - Deaths expected in 2012 had CHD mortality rates in 1990 persisted (***Poisson distribution***) |  |
| **Risk factors** | | |
| Prevalence/mean estimates (pooled data; national estimates for) | - Prevalence estimates (smoking, physical activity, diabetes): (***Beta distribution***: cases, sample-size minus cases) - Continuous variables (Body Mass Index, SBP, total cholesterol, fruit and vegetable consumption): (***Normal distribution***: mean, SE of mean) |  |
| RR: **smoking** | ***Ersatz RR function*** (RR, SE ln(RR)):  RRs and 95% CIs shown in Table A3 | Ezzati et al (2005) [57] |
| RR: **physical activity** | ***Ersatz RR function*** (RR, SE ln(RR)):  RRs and 95% CIs shown in Table A3 | Bull et al (2004) [58] |
| RR: **diabetes** | ***Ersatz RR function*** (RR, SE ln(RR)):  RRs and 95% CIs shown in Table A2. | Roglic and Unwin (2010) [10]; Huxley et al (2006) [11] |
| Beta coefficient: **Body Mass Index** | ***Normal distribution*** (mean, SE of mean): Table A2 | Bogers et al (2006) [55], James et al (2004) [56]. Parameters on the log scale. |
| Beta coefficient: **SBP** | ***Normal distribution*** (mean, SE of mean): Table A2 | Prospective studies collaborative meta-analysis (2002) [53]. Parameters on the log scale. |
| Beta coefficient: **total cholesterol** | ***Normal distribution*** (mean, SE of mean): Table A2 | Prospective studies collaborative meta-analysis (2007) [54]. Parameters on the log-scale. |
| Beta coefficient: **Fruit and vegetable consumption** | ***Normal distribution*** (mean, SE of mean): Table A2 | Dauchet et al (2006) [7]. Parameters on the log-scale. |
| **ST elevation myocardial infarction (STEMI)** | | |
| **Eligible patients**:  Emergency admissions with a primary diagnosis of myocardial infarction (ratio of STEMI/nSTEMI as 40/60) | ***Poisson distribution*** (admissions) |  |
| **Case fatality rate** | Sample size (*n*) = STEMI admissions:  ***Beta distribution*** (cases = *n* × CFR estimate, non-cases = *n* – cases) |  |
| **Treatment uptake** | - *Medications and in-hospital CPR:* ***Beta distribution*** (cases = STEMI admissions from BNR × medication uptake, non-cases = STEMI admissions – cases) - *PCI and CABG*: ***Beta distribution*** (cases = MI admissions from HES × PCI/CABG uptake, non-cases = MI admissions – cases) |  |
| **Relative risk reduction:** | ***Ersatz RR function*** (RRR, SE ln(RRR)): |  |
| In-hospital CPR | M & F (33%,0.103): absolute risk reduction |  |
| Thrombolysis  Aspirin  Beta-blockers  Primary PCI  Primary CABG surgery  ACE Inhibitors | M & F (0.31,0.298)  M & F (0.23,0.177)  M & F (0.04,0.691): assumed lower limit of 1%  M & F (0.30,0.587)  M & F (0.39,0.293)  M & F (0.07,0.435) |  |
| Clopidogrel | M & F (0.03,0.457) |  |
| **Non-ST segment elevation acute coronary syndrome (NSTEACS)** | | |
| **Eligible patients**:  Emergency admissions with a primary diagnosis of myocardial infarction (ratio of STEMI/nSTEMI as 40/60) or primary diagnosis of unstable angina | ***Poisson distribution*** (nSTEMI + unstable angina admissions) |  |
| **Case fatality rate** | Sample size (*n*) = nSTEMI + unstable angina admissions:  ***Beta distribution*** (cases = *n* × CFR estimate, non-cases = *n* – cases) | Wijeysundera et al (2010) [5] |
| **Treatment uptake** | - *Medications and in-hospital CPR:* ***Beta distribution*** (cases = NSTEACS admissions × medication uptake, non-cases = NSTEACS admissions – cases) - *PCI and CABG*: ***Beta distribution*** (cases = unstable angina admissions from HES × PCI/CABG uptake, non-cases = unstable angina admissions – cases) |  |
| **Relative risk reduction:** | ***Ersatz RR function*** (RRR, SE ln(RRR)): |  |
| In-hospital CPR | M & F (33%,0.103): absolute risk reduction | Tunstall-Pedoe (1992) [33] |
| Aspirin & heparin  Primary CABG surgery  Early PCI  Beta blockers  Clopidogrel  ACE Inhibitors | M & F (0.33,0.470)  M & F (0.39,0.293)  M & F (0.32,0.592)  M & F (0.04,0.691): assumed lower limit of 1%  M & F (0.07,0.435)  M & F (0.07,0.435) | Oler (1996) [36]  Yusuf (1994) [27]  RITA 3 (Fox 2005) [38]  Freemantle (1999) [29]  Yusuf (2001) [39]  ACE-I MI Collaborative Group (1998) [40] |
| Aspirin alone | M & F (0.15,0.139) | Antithrombotic Trialists’ Collaboration ATC (2002) [35] |
| Platelet glycoprotein IIB/IIIA inhibitors | M & F (0.09,0.530) | Boersma (2002) [37] |
| **Secondary prevention post myocardial infarction (MI)** | | |
| **Eligible patients**:  Ever having had a myocardial infarction (prior to 1/1/2007) | ***Poisson distribution*** (Population in 2012 × (post-MI prevalence obtained from GPRD) minus assumed overlap with Heart Failure) |  |
| **Case fatality rate** | Sample size (*n*) = ever having had MI in GPRD in 2007:  ***Beta distribution*** (cases = *n* × CFR estimate, non-cases = *n* – cases) | Wijeysundera et al (2010) [5] |
| **Treatment uptake** | ***Beta distribution*** (cases = *n* × medication uptake, non-cases = *n* – cases) | GPRD (2000 and 2007 for start and end year respectively) |
| **Compliance** | Sample size (*n1*) = ever having had MI in GPRD in 2007 with record of medication use:  ***Beta distribution*** (cases = *n1* × assumed compliance, non-cases = *n1* – cases) |  |
| **Relative risk reduction:** | ***Ersatz RR function*** (RRR, SE ln(RRR)): |  |
| Aspirin  Beta blockers  ACE Inhibitors  Statins  Warfarin | M & F (0.15,0.139)  M & F (0.23,0.185)  M & F (0.20,0.177)  M & F (0.24,0.245)  M & F (0.22,0.305) | ATC (2002) [35]  Freemantle (1999) [29]  Flather (2000) [40]  Hulten (2006) [41]  Anand and Yusuf (1999) [42] |
| **Secondary prevention post revascularisation** | | |
| **Eligible patients**:  Ever having had a revascularisation procedure, and assumed be alive prior to 1/1/2007 | - ***Poisson distribution*** (CABG/PTCA procedures from 2000 to 2007 minus i) annual 5% mortality adjustment, ii) assumed overlap with post myocardial infarction group) - ***Rehabilitation (mortality benefits within last 5 years only): Poisson distribution*** (CABG/PTCA procedures from 2002 to 2007 minus i) annual 5% mortality adjustment, ii) assumed overlap with post myocardial infarction group) | Hospital Episode Statistics (HES) |
| **Case fatality rate** | Sample size (*n*) = ever having had post-revascularisation in GPRD in 2007:  ***Beta distribution*** (cases = *n* × CFR estimate, non-cases = *n* – cases) | Wijeysundera et al (2010) [5] |
| **Treatment uptake** | ***Beta distribution*** (cases = *n* × medication uptake, non-cases = *n* – cases) |  |
| **Compliance** | Sample size (*n1*) = ever having had revascularisation in GPRD in 2007 with record of medication use:  ***Beta*** ***distribution*** (cases = *n1* × assumed compliance, non-cases = *n1* – cases) |  |
| **Relative risk reduction:** | ***Ersatz RR function*** (RRR, SE ln(RRR)): |  |
| Aspirin  Beta blockers  ACE Inhibitors  Statins  Rehabilitation  Warfarin | M & F (0.15,0.139)  M & F (0.23,0.185)  M & F (0.20,0.177)  M & F (0.24,0.245)  M & F (0.26,0.347)  M & F (0.22,0.305) | ATC (2002) [35]  Freemantle (1999) [29]  Flather (2000) [40]  Hulten (2006) [41]  Taylor (2004) [43]  Anand and Yusuf (1999) [42] |
| **Chronic stable coronary artery disease** | | |
| **Eligible patients**:  Ever having had chronic stable artery disease but no myocardial infarction (prior to 1/1/2007) | ***Poisson*** ***distribution*** (Population in 2007 × (angina but no myocardial infarction prevalence obtained from GPRD) minus i) emergency admissions for unstable angina and ii) assumed overlaps with heart failure and post-revascularisation groups) | General Practice Research Database (GPRD) |
| **Case fatality rate** | Sample size (*n*) = ever having had angina but no MI in GPRD in 2007:  ***Beta distribution*** (cases = *n* × CFR estimate, non-cases = *n* – cases) | Wijeysundera et al (2010) [5] |
| **Treatment uptake** | ***Beta distribution*** (cases = *n* × medication uptake, non-cases = *n* – cases) | GPRD (2000 and 2007 for start and end year respectively) |
| **Compliance** | Sample size (*n1*) = ever having had angina but no MI in GPRD in 2007 with record of medication use  ***Beta distribution*** (cases = *n1* × assumed compliance, non-cases = *n1* – cases) |  |
| **Relative risk reduction:** Statins  Aspirin  ACE Inhibitors | ***Ersatz RR function*** (RRR, SE ln(RRR)):  M & F (0.23,0.244)  M & F (0.15,0.139)  M & F (0.17,0.177) | Wilt (2004) [45]  ATC (2002) [35]  Al-Mallah (2006) [46] |
| **CABG for chronic stable coronary artery disease (0-5 years)** | | |
| **Eligible patients**:  Ever having had chronic stable artery disease but no myocardial infarction (prior to 1/1/2007) | ***Poisson distribution*** (estimated count of patients with stable coronary artery disease (described above) in 2007 × (estimated uptake of CABG)):   - ***2007***: Uptake of CABG: (number of CABG procedures from 2002 to 2007)/eligible patients in 2007 - ***2000***: Uptake of CABG: (number of CABG procedures from 1995 to 2000)/eligible patients in 2000 | Hospital Episode Statistics |
| **Case fatality rate** | As described in the post-revascularisation group |  |
| **Treatment uptake and compliance** | Fixed at 100% |  |
| **Relative risk reduction:** CABG (0-5 years) | ***Ersatz RR function*** (RRR, SE ln(RRR)):  M & F (0.39,0.293) | Yusuf (1994) [27] |
| **CABG for chronic stable coronary artery disease (6-10 years)** | | |
| **Eligible patients**:  Ever having had chronic stable artery disease but no myocardial infarction (prior to 1/1/2007) | ***Poisson distribution*** (estimated count of patients with stable coronary artery disease (described above) in 2007 × (estimated uptake of CABG)):   - ***2007***: Uptake of CABG: (number of CABG procedures from 2000 & 2001)/eligible patients in 2007 - ***2000***: Uptake of CABG: (number of CABG procedures from 1993 & 1994)/eligible patients in 2000 | Hospital Episode Statistics |
| **Case fatality rate** | As described in the post-revascularisation group |  |
| **Treatment uptake and compliance** | Fixed at 100% |  |
| **Relative risk reduction:** CABG (6-10 years) | ***Ersatz RR function*** (RRR, SE ln(RRR)):  M & F (0.32,0.243) | Yusuf (1994) [27] |
| **Heart failure in patients requiring hospitalisation** | | |
| **Eligible patients**:  Admissions with a primary diagnosis of heart failure | ***Poisson distribution*** (admissions minus 50% assumed not to be due to CHD) | Hospital Episode Statistics (HES) |
| **Case fatality rate** | Sample size (*n*) = HES admissions for HF divided by 2 as only half assumed to be CHD related  ***Beta distribution*** (cases = *n* × CFR estimate, non-cases = *n* – cases) | Wijeysundera et al (2010) [5] |
| **Treatment uptake** | - Aspirin: as described in the post-myocardial infarction group   Other medications: Sample size (*n1*) = HF admissions from NHS survey:   - ***Beta distribution*** (cases = *n1* × medication uptake, non-cases = *n1* – cases) | NHS Heart Failure survey (2005).  2005 rates taken as 2007 values. Rates assumed 10% lower in 2000. |
| **Compliance** | Sample size (*n2*) = HF admissions from NHS survey with record of medication use:  ***Beta distribution*** (cases = *n2* × assumed compliance, non-cases = *n2*  – cases) | NHS Heart Failure Survey |
| **Relative risk reduction:** Aspirin  ACE Inhibitors  Beta blockers  Spironolactone | ***Ersatz RR function*** (RRR, SE ln(RRR)):  M & F (0.15,0.139)  M & F (0.20,0.177)  M & F (0.35,0.128)  M & F (0.30,0.128) | ATC (2002) [35]  Flather (2000) [40]  Shibata (2001) [47]  Pitt (1999) [48] |
| **Heart failure in the community** | | |
| **Eligible patients**:  Ever having had heart failure (prior to 1/1/2007) | ***Poisson distribution*** (Population in 2007 × HF prevalence obtained from GPRD divided by 2 as only half assumed to be CHD related) minus HF hospital admissions) | General Practice Research Database, Hospital Episode Statistics |
| **Case fatality rate** | Sample size (*n*) = ever having had HF in GPRD in 2007:  ***Beta distribution*** (cases = *n* × CFR estimate, non-cases = *n* – cases) | Wijeysundera et al (2010) [5] |
| **Treatment uptake** | ***Beta distribution*** (cases = *n* × medication uptake, non-cases = *n* – cases) |  |
| **Compliance** | Sample size (*n1*) = ever having had HF in GPRD in 2007 with record of medication use:  ***Beta distribution*** (cases = *n1* × assumed compliance, non-cases = *n1* – cases) |  |
| **Relative risk reduction:** Aspirin  ACE Inhibitors  Beta blockers  Spironolactone | ***Ersatz RR function*** (RRR, SE ln(RRR)):  M & F (0.15,0.139)  M & F (0.20,0.177)  M & F (0.35,0.128)  M & F (0.31,0.216) | ATC (2002) [35]  Flather (2000) [40]  Shibata (2001) [47]  Pitt (1999) [48] |
| **Primary prevention therapies: Statins** | | |
| **Eligible patients**:  Population | Population counts (no error) | Office for National Statistics |
| **Treatment uptake** | % never having had angina or heart attack and currently taking lipid lowering drugs prescribed by a doctor: (***Beta distribution***: cases, sample-size minus cases) | Health Survey for England |
| **Case fatality rate** | Sample size (*n*) = never having had angina or heart attack and currently taking lipid lowering drugs in 2006:  ***Beta distribution*** (cases = *n* × CFR estimate, non-cases = *n* – cases) | Wijeysundera et al (2010) [5] |
| **Compliance** | ***Beta distribution*** (cases = *n* × assumed compliance, non-cases = *n* – cases) | Health Survey for England |
| **Relative risk reduction:** Statins | ***Ersatz RR function*** (RRR, SE ln(RRR)):  M & F (0.35,0.396) | Pignone (2000) [52] |
| **Primary prevention therapies: Treatments for high blood pressure** | | |
| **Eligible patients**:  Population | Population counts (no error) | Office for National Statistics |
| **Treatment uptake** | % never having had angina or heart attack and currently taking medication specifically prescribed to treat high blood pressure: (***Beta distribution***: cases, sample-size minus cases) | Health Survey for England |
| **Case fatality rate** | Sample size (*n*) = never having had angina or heart attack and currently taking medication to lower blood pressure in 2006:  ***Beta distribution*** (cases = *n* × CFR estimate, non-cases = *n* – cases) | Wijeysundera et al (2010) [5] |
| **Compliance** | ***Beta distribution*** (cases = *n* × assumed compliance, non-cases = *n* – cases) | Health Survey for England |
| **Relative risk reduction:** Treatments for high blood pressure | ***Ersatz RR function*** (RRR, SE ln(RRR)):  M & F (0.13,0.294) | Law (2003) [51] |
