# Supplementary material for: Adverse risk factor trends limit gains in coronary heart disease mortality in Barbados: 1990-2012
Source: PLoS One. 2019 Apr 17;14(4):e0215392. doi: 10.1371/journal.pone.0215392 (PMC6469800; doi:10.1371/journal.pone.0215392)
Supplement: S1 Appendix — (DOCX) [file pone.0215392.s008.docx]

# S1 Appendix: Management of Coronary Heart Disease - Interview guide

Introduction: Thank you for agreeing to participate in this exercise to assist us in determining key information regarding the management of acute myocardial infarction and unstable angina in Barbados in 1990.

Interview guide (Semi-structured interview)

1. What was your occupation during the years 1989 - 1992?

Prompts for memory: 1990 was the year Nelson Mandela was released from prison, do you remember what your mode of employment was at that time?

1. Where was your main place of employment during the years 1989 to 1992?

Prompts: Queen Elizabeth Hospital, Private Hospital, Private Ambulatory clinic, Public Ambulatory clinic, Central Ministry of Health, Barbados Drug Service

1. Where was your secondary place of employment for the years 1989 to 1992?

Prompts: Queen Elizabeth Hospital, Private Hospital, Private Ambulatory clinic, Public Ambulatory clinic, Central Ministry of Health, Barbados Drug Service

1. Which of the following drugs were used in the in hospital management of an acute myocardial infarction in 1989-1992?

| Drug |  |  |  | If yes, state approximate proportion of patients receiving drug post MI | If yes, provide an approximation for compliance |
| --- | --- | --- | --- | --- | --- |
| Statins | Yes | No | Not sure/Cannot recall |  |  |
| Aspirin | Yes | No | Not sure/Cannot recall |  |  |
| Aspirin & Heparin | Yes | No | Not sure/Cannot recall |  |  |
| Warfarin | Yes | No | Not sure/Cannot recall |  |  |
| ACE inhibitors | Yes | No | Not sure/Cannot recall |  |  |
| Beta-blockers | Yes | No | Not sure/Cannot recall |  |  |
| Spironolactone | Yes | No | Not sure/Cannot recall |  |  |
| PG IIIa/IIB | Yes | No | Not sure/Cannot recall |  |  |
| Thrombolysis | Yes | No | Not sure/Cannot recall |  |  |
| Gemfibrozil | Yes | No | Not sure/Cannot recall |  |  |
| Niacin | Yes | No | Not sure/Cannot recall |  |  |

1. Regarding the above drugs, did your prescribing practice vary if the patient was younger or older?
2. Regarding the above drugs, did your prescribing practice vary if the patient was male or female?
3. Which of the following procedures were used in the hospital management of acute myocardial infarction in 1989-1992?

| PTCA | Yes | No | Not sure/Cannot recall |
| --- | --- | --- | --- |
| Rehabilitation | Yes | No | Not sure/Cannot recall |
| CPR in community | Yes | No | Not sure/Cannot recall |
| CPR in hospital | Yes | No | Not sure/Cannot recall |
| CABG | Yes | No | Not sure/Cannot recall |

1. Which of the following drugs were used in the in hospital management of unstable angina in 1989-1992??

| Drug |  |  |  | If yes, state approximate proportion of patients receiving drug post MI | If yes, provide an approximation for compliance |
| --- | --- | --- | --- | --- | --- |
| Statins | Yes | No | Not sure/Cannot recall |  |  |
| Aspirin | Yes | No | Not sure/Cannot recall |  |  |
| Aspirin & Heparin | Yes | No | Not sure/Cannot recall |  |  |
| Warfarin | Yes | No | Not sure/Cannot recall |  |  |
| ACE inhibitors | Yes | No | Not sure/Cannot recall |  |  |
| Beta-blockers | Yes | No | Not sure/Cannot recall |  |  |
| Spironolactone | Yes | No | Not sure/Cannot recall |  |  |
| PG IIIa/IIB | Yes | No | Not sure/Cannot recall |  |  |

1. Regarding the above drugs, did your prescribing practice vary if the patient was younger or older?
2. Regarding the above drugs, did your prescribing practice vary if the patient was male or female?

During the interview, each of these questions will be repeated for the time periods 1999 to 2001 and 2008 to 2010. The prompts used will be appropriate for the time period being examined.
