# Supplementary material for: Adverse risk factor trends limit gains in coronary heart disease mortality in Barbados: 1990-2012
Source: PLoS One. 2019 Apr 17;14(4):e0215392. doi: 10.1371/journal.pone.0215392 (PMC6469800; doi:10.1371/journal.pone.0215392)
Supplement: S2 Appendix — (DOCX) [file pone.0215392.s009.docx]

# S2 Appendix: Methodology and results related to documentary analysis and semi-structured interviews.

**Introduction**

In this study we seek to document the historical development of the management of acute coronary syndromes (primarily myocardial infarction and unstable angina) in a small island developing state. The time period examined was primarily during the decade of the 1990s. The information collected will be used within an analytical model to explain the relative contributions of risk factors changes and medical treatments to declines in cardiovascular mortality over that time.

**Methods**

Phase one

Documentary analysis was conducted by reviewing all Chief Medical Officer Reports and Barbados National Drug Formularies printed during the period 1988 to 2012. All the drugs known to be effective for prevention and control of acute coronary syndrome and which were used in the coronary heart disease IMPACT model developed by Capewell et al^29,30^ were searched for in each formulary to determine their availability to the majority of Barbadians in the 1990s and early 2000s. Since most drugs used to treat hypertension, myocardial infarction and strokes were provided free of cost to patients in the private and public sector from 1990 to 2012, lack of availability of the drug in the national formulary was considered a proxy for assumed low usage. The converse however was not held to be true.

Phase two

Health care professionals- cardiologists, internists and accident and emergency specialists were invited to take part in an interview process. A purposive sample of eight persons was chosen to obtain the necessary information. Persons chosen needed to have been practicing and managing myocardial infarctions actively during the period 1990 to 2012. A semi-structured interview was conducted with each participant using a life grid approach. In this approach, participants are asked to recall historical events and behaviours by using significant temporal references of external or personal (eg. family or work-related events) to help the participant remember the information of interest with greater accuracy. The life grid approach has been used in epidemiological studies requiring dating and recall of temporally distant exposures ^31-33^. The life grid interview for this study required that participants remember what was occurring in their personal and professional lives in the year 1990. They were asked to recall their employment status and job title at the time of significant local and international events. They were then asked about the availability of specific drugs and procedures in Barbados at the time of interest (Appendix A).

Interviews were recorded with the permission of the participants and transcribed verbatim. If participants refused to be recorded, only notes were taken of the interview. Data from the interviews were analysed and in cases where there was significant disagreement amongst participants about a particular question, interviewees were asked to review their initial answer in the light of what has been proposed by others. This attempt to reach consensus amongst experts is characteristic of the Delphi process. There were however no significant disparities in information provided by participants. Data was reviewed to answer specific questions regarding availability of specific drugs and procedures in the decade of the 1990s. Having obtained this, transcripts were reviewed and coded to determine if any themes related to the treatment context emerged.

This study received ethical approval from the University of the West Indies/Ministry of Health Institutional Review Board.

**Results**

Historical impact of Barbados Drug Service regulations of treatment availability

In 1980, the Barbados Drug Service (BDS) was established with the main goal of reducing the cost of prescribed drugs while ensuring the continuous availability of quality products of known therapeutic effectiveness. A Special Benefit Service was later added (1986) which provides medication free of cost to all patients seeking care at one of eight primary care centres (polyclinics). This includes persons being treated for non-communicable disease such as hypertension, diabetes and coronary heart disease. For the period 1990-2011, persons being managed in the private sector were allowed to access essential drugs identified on the formulary free of charge. Beginning in 2011, individuals using private pharmacies to access drugs are required to pay a dispensing fee even though the actual cost of the drug remained free.

The earliest formulary reviewed was that which documented the available drugs for the year 1988-1989. Several drugs used in the IMPACT model, warfarin , aspirin, spironolactone and beta-blockers were all available in 1990(Table B1) as well as in 2012. The beta-blockers on formulary were atenolol, metoprolol and propanolol. ACE inhibitors first became available on the formulary and the special benefits drugs list in 1991/1992. Statins were added to the formulary in 1996/1997. At that time only Fluvastatin(Lescol) was added. Gemfibrozil and niacin were added in 1998-1999 and 2011-2012 respectively. Angiotensin Receptor Blockers became available on formulary in 2002 but could only be prescribed with authorization from Consultant Physicians. In the 2003-2004 edition, the ARBs losartan, telmisartan and valsartan were made more widely available with prescriptions allowed from all categories of physicians.

# Table B1: Availability of CHD drugs of interest for the period 1988 to 2000

| Drugs of interest | On in 1988 | On in 1990-91 | On in 1991-92 |
| --- | --- | --- | --- |
| Warfarin | Yes | Yes | Yes |
| Aspirin | Yes | Yes | Yes |
| Spironolactone | Yes | Yes | Yes |
| ACE Inhibitors | No | No | Yes |
| Beta-blockers | Yes | Yes | Yes |
| Gemfibrozil | No | No | No |
| Niacin | No | No | No |
| Statins | No | No | No |
| GTN/Isosorbide Dinitrate | Yes | Yes | Yes |
| Cholestyramine | Yes | Yes | Yes |

Documentary analysis also revealed that Barbados Drug service dispensing for certain MI treatment related drugs more than doubled from the years 2000 to 2005 to 2010(Table B2). The notable exception to this can be seen for ACE inhibitor dispensing which increased from 2000 to 2005 but decreased in 2010. This was likely due to the introduction of ARBs onto the formulary in 2003.

# Table B2: Trends in drugs prescribed for the period 2000 to 2010

|  | **ACE INHIBITORS** | | | **STATINS** | | | **ASPIRIN** | | |
| --- | --- | --- | --- | --- | --- | --- | --- | --- | --- |
|  | **2000** | **2005** | **2010** | **2000** | **2005** | **2010** | **2000** | **2005** | **2010** |
| **TOTAL** | **5,345** | **6,674** | **4,498** | **1,593** | **6,223** | **9,323** | **1,482** | **2,753** | **7,513** |
|  | **BETA BLOCKERS** | | | **SPIRONOLACTONE** | | | **WARFARIN** | | |
|  | **2000** | **2005** | **2010** | **2000** | **2005** | **2010** | **2000** | **2005** | **2010** |
| **TOTAL** | **2,798** | **3,602** | **5,175** | **40** | **117** | **227** | **61** | **81** | **239** |

**Timeline for the introduction of key protocols and procedures used in the management of AMI**

Interviews with health care professionals to identify key timepoints for the introduction of various ACS related treatments were conducted to the point of saturation, which was at six interviews. Below we present the emerging themes.

History of the Cardiac Unit

In the early 1970s the Cardiac Outpatients Unit was opened at the Queen Elizabeth Hospital and served both the Barbadian and Eastern Caribbean populations well. The Cardiac Unit is an outpatient department managed by cardiologists providing expert care in the full complement of cardiac conditions with particular services for coronary heart disease. The unit gradually became equipped with cardiac diagnostic equipment necessary for the monitoring and management of heart disease. By 1990 the unit provided non-invasive diagnostic procedures such as electrocardiograms, treadmill stress tests and echocardiographic services^34^. In the 1990s, there were significantly more modalities added with less development noted in the 21^st^ century (Table B1). By the year 2012, Barbados had successfully introduced many of the medical and surgical therapies used in the management of acute coronary syndromes in more developed regions of the world (Table B3).

A few critical therapeutic modalities remained unavailable in 2012, the most striking of which is the lack of the availability of percutaneous coronary intervention (PCI) in the public sector. This service has since been added (2015) but as of December 2016 was not yet offered in the acute setting for myocardial infarctions. In the acute setting, patients with ST segment elevation MI are treated with thrombolytics if they meet the criteria for this group of drugs.

Interviewees noted that we have been generally up to date on our pharmacological interventions but lamented not having a Coronary Care Unit (CCU) and a standard Advanced Cardiac Life Support (ACLS) team to respond to cardiac arrest situations. The CCU would be an inpatient service dedicated to the care of patients who need a higher level of care than normal after acute heart-related illnesses, such as a heart attack.

# Table B3 Introduction of key protocols for the management of acute coronary syndrome

| Year |  |
| --- | --- |
| 1990 | Aspirin, Beta-blockers, Spironolactone, Warfarin already available |
| 1991 | CPR training begins/ACLS and BLS programmes |
| 1992 | ACE inhibitors introduced on formulary |
| 1993 | Cardiac Catheter Laboratory Reintroduced after 5 year dormancy period/Thrombolytic therapy with Streptokinase now available |
| 1994 | Coronary Artery Bypass Grafting begins |
| 1997 | Statins(Fluvastatin) introduced on formulary |
| 2002 | Angiotensin Receptor Blockers introduced as Specially Administered Drugs(Requiring Consultant Authorization) |
| 2003 | Angiotension Receptor Blockers added on full formulary |
| 2011 | Thrombolytic therapy with Metalyse now available |

Barriers and Facilitators of the development of Acute Coronary Syndrome Management in Barbados

Cost was a persistent barrier to the introduction of new medical therapies and procedures.

When asked about perceived barriers to introduction of new medical therapies and procedures for management of acute coronary syndromes and its complications, cost was a persistent factor.

“Price was always the issue,” noted one participant.

Another participant noted that initially statins were introduced into the private sector while its public sector introduction was delayed by at least one year in part due to economic considerations. Cost extends itself into staffing shortages and lack of equipment maintenance. Often maintenance of the cardiac unit is sacrificed due to lack of finances. This lack of maintenance leads to destruction and thus unavailability of critical equipment.
