# Supplementary material for: Adverse risk factor trends limit gains in coronary heart disease mortality in Barbados: 1990-2012
Source: PLoS One. 2019 Apr 17;14(4):e0215392. doi: 10.1371/journal.pone.0215392 (PMC6469800; doi:10.1371/journal.pone.0215392)
Supplement: S3 Appendix — (DOCX) [file pone.0215392.s010.docx]

# S3: Appendix: Parameter Estimates

Table 1. Parameter estimates for the deaths expected in 2012 had the rates remained the same as 1990

Poisson

|  | Deaths to be explained (count) |
| --- | --- |
| M 25-34 | 0 |
| M 35-44 | 3 |
| M 45-54 | 14 |
| M 55-64 | 14 |
| M 65-74 | 10 |
| M 75-84 | 15 |
| M 85+ | 6 |
| F 25-34 | 0 |
| F 35-44 | 2 |
| F 45-54 | 2 |
| F 55-64 | 9 |
| F 65-74 | 17 |
| F 75-84 | 27 |
| F 85+ | 19 |
| M | 63 |
| F | 76 |
| M+F | 139 |

Table 2: Risk factor parameters – continuous variables

|  | SBP (log) |  | Cholesterol |  | BMI |  | F&V |  |
| --- | --- | --- | --- | --- | --- | --- | --- | --- |
|  | BETA MEAN | BETA SE | BETA MEAN | BETA SE | BETA MEAN | BETA SE | BETA MEAN | BETA SE |
| M 25-34 | 0.036 | 0.004 | 0.80 | 0.081 | 0.036 | 0.005 | 0.04 | 0.016 |
| M 35-44 | 0.036 | 0.004 | 0.80 | 0.081 | 0.036 | 0.005 | 0.04 | 0.016 |
| M 45-54 | 0.035 | 0.003 | 0.76 | 0.077 | 0.030 | 0.004 | 0.04 | 0.016 |
| M 55-64 | 0.032 | 0.003 | 0.45 | 0.046 | 0.023 | 0.003 | 0.04 | 0.016 |
| M 65-74 | 0.027 | 0.002 | 0.24 | 0.024 | 0.015 | 0.002 | 0.04 | 0.016 |
| M 75-84 | 0.021 | 0.002 | 0.12 | 0.012 | 0.012 | 0.002 | 0.04 | 0.016 |
| M 85+ | 0.016 | 0.002 | 0.08 | 0.009 | 0.010 | 0.001 | 0.04 | 0.016 |
| F 25-34 | 0.046 | 0.005 | 0.84 | 0.086 | 0.036 | 0.005 | 0.04 | 0.016 |
| F 35-44 | 0.046 | 0.005 | 0.84 | 0.086 | 0.036 | 0.005 | 0.04 | 0.016 |
| F 45-54 | 0.046 | 0.005 | 0.73 | 0.075 | 0.030 | 0.004 | 0.04 | 0.016 |
| F 55-64 | 0.035 | 0.004 | 0.43 | 0.044 | 0.023 | 0.003 | 0.04 | 0.016 |
| F 65-74 | 0.032 | 0.003 | 0.26 | 0.027 | 0.015 | 0.002 | 0.04 | 0.016 |
| F 75-84 | 0.026 | 0.003 | 0.17 | 0.018 | 0.012 | 0.002 | 0.04 | 0.016 |
| F 85+ | 0.019 | 0.002 | 0.05 | 0.005 | 0.010 | 0.001 | 0.04 | 0.016 |

Table 3: Risk factor paramaters (Categorical variables)

|  | Smoking |  |  |  |
| --- | --- | --- | --- | --- |
|  | PAR | 95%LCL | 95%UCL | ln(SE) |
| M 25-34 | 5.510 | 2.47 | 12.25 | 0.408 |
| M 35-44 | 5.510 | 2.47 | 12.25 | 0.408 |
| M 45-54 | 3.040 | 2.66 | 3.48 | 0.069 |
| M 55-64 | 2.513 | 2.22 | 2.84 | 0.063 |
| M 65-74 | 1.691 | 1.52 | 1.89 | 0.056 |
| M 75-84 | 1.310 | 1.11 | 1.56 | 0.088 |
| M 85+ | 1.050 | 0.78 | 1.43 | 0.155 |
| F 25-34 | 2.260 | 0.83 | 6.14 | 0.510 |
| F 35-44 | 2.260 | 0.83 | 6.14 | 0.510 |
| F 45-54 | 3.780 | 3.10 | 4.62 | 0.102 |
| F 55-64 | 3.212 | 2.70 | 3.82 | 0.089 |
| F 65-74 | 2.166 | 1.89 | 2.47 | 0.067 |
| F 75-84 | 1.580 | 1.33 | 1.88 | 0.087 |
| F 85+ | 1.380 | 1.08 | 1.77 | 0.126 |

| Physical activity |  |  |  | Diabetes |  |  |  |
| --- | --- | --- | --- | --- | --- | --- | --- |
| PAR | 95%LCL | 95%UCL | ln(SE) | PAR | 95%LCL | 95%UCL | ln(SE) |
| 1.50 | 1.35 | 1.67 | 0.040 | 3.08 | 3.47 | 5.20 | 0.10 |
| 1.50 | 1.35 | 1.67 | 0.040 | 4.60 | 2.58 | 3.86 | 0.10 |
| 1.50 | 1.35 | 1.67 | 0.040 | 2.80 | 1.71 | 2.57 | 0.10 |
| 1.50 | 1.35 | 1.67 | 0.071 | 2.00 | 1.59 | 2.39 | 0.10 |
| 1.44 | 1.30 | 1.61 | 0.071 | 1.65 | 1.49 | 2.23 | 0.10 |
| 1.32 | 1.19 | 1.47 | 0.071 | 1.40 | 1.37 | 2.05 | 0.10 |
| 1.23 | 1.11 | 1.37 | 0.071 | 1.40 | 1.37 | 2.05 | 0.10 |
| 1.50 | 1.35 | 1.68 | 0.093 | 3.20 | 6.04 | 9.06 | 0.10 |
| 1.50 | 1.35 | 1.68 | 0.093 | 3.10 | 4.51 | 6.76 | 0.10 |
| 1.50 | 1.35 | 1.68 | 0.093 | 2.86 | 3.05 | 4.57 | 0.10 |
| 1.50 | 1.35 | 1.68 | 0.093 | 2.60 | 2.50 | 3.74 | 0.10 |
| 1.45 | 1.30 | 1.61 | 0.103 | 2.10 | 2.04 | 3.06 | 0.10 |
| 1.33 | 1.20 | 1.47 | 0.103 | 1.60 | 1.89 | 2.83 | 0.10 |
| 1.24 | 1.13 | 1.37 | 0.103 | 1.60 | 1.89 | 2.83 | 0.10 |
